# Supervised Fine-Tuning of Large Language Models With Chain-of-Thought Reasoning for Pediatric Heart Disease Detection in Unstructured Echocardiogram Reports: Algorithm Development and Validation

**DOI:** 10.2196/90968

**Published:** 2026-06-08

**Authors:** Haoming Shi, Justin B Long, Michael C Fiedorek, Hannah D Kilday, Henry P Foote, Christoph P Hornik, Aditya Nagori, Yifan Xiang, Rishikesan Kamaleswaran

**Affiliations:** 1 Department of Biomedical Engineering, Duke University, 1427 Fitzpatrick Center Box 90281, Durham, NC, 27708, United States, 1 9196695131; 2 Department of Surgery, Duke University School of Medicine, Durham, NC, United States; 3 Department of Anesthesiology, Duke University School of Medicine, Durham, NC, United States; 4 Department of Anesthesiology, Emory University School of Medicine, Atlanta, GA, United States; 5 Department of Anesthesiology, Children’s Healthcare of Atlanta, Atlanta, GA, United States; 6 Department of Pediatrics, Duke University School of Medicine, Durham, NC, United States

**Keywords:** congenital heart defects, echocardiogram, machine learning, large language model, natural language processing

## Abstract

**Background:**

Pediatric heart disease (PHD), including congenital heart defects, is often incompletely captured in electronic health records, particularly when clinical significance must be inferred from unstructured echocardiogram reports. Automated methods capable of extracting clinically meaningful PHD from narrative reports could improve clinical decision support and research applications.

**Objective:**

The aim of the study is to evaluate the feasibility of using supervised fine-tuning of large language models (LLMs), with and without chain-of-thought (CoT) reasoning, to characterize patients with clinically significant or historical PHD from unstructured echocardiogram reports.

**Methods:**

We developed a PHD detection algorithm using fine-tuned open-source LLMs, including LLaMA (Meta) and Qwen (Alibaba), to analyze 9749 echocardiogram reports. A subset of 712 reports was adjudicated by 2 pediatric cardiac anesthesiologists, classifying 506 (71.1%) as clinically significant PHD and 206 (28.9%) as not significant. While DeepSeek R1 has shown improved performance with CoT reasoning, its application in medical contexts is underexplored. We incorporated R1-generated CoT into model prompts and fine-tuned backbone LLMs.

**Results:**

The fine-tuned Qwen-7B-10k-overthink-CoT achieved the highest accuracy (92.4%), outperforming Qwen-7B-without-CoT (90%), LLaMA-3B-without-CoT (87.9%), Qwen-3B-without-CoT (85.6%), Qwen-3B-10k-overthink-CoT (68.5%), and LLaMA-3B-10k-overthink-CoT (46.2%). In a second dataset, an external validation was performed (n=113; 64 positive, 49 negative), Qwen-7B-10k-overthink-CoT sustained a strong, balanced performance (82.7%), followed by Qwen-7B-without-CoT (88.4%), LLaMA-3B-without-CoT (86.8%), Qwen-3B-without-CoT (84.5%), Qwen-3B-10k-overthink-CoT (58.9%), and LLaMA-3B-10k-overthink-CoT (46.2%). The fine-tuned Qwen-7B model with overthinking CoT (10,000 tokens) achieved the highest internal accuracy (92.4%), with balanced sensitivity and specificity. Across repeated runs, CoT-enhanced models demonstrated improved classification consistency compared to non-CoT models (Qwen-7B-without-CoT: 90%, LLaMA-3B-without-CoT: 87.9%, Qwen-3B-without-CoT: 85.6%). In external validation (n=113), non-CoT variants achieved higher accuracy (up to 88.4%), whereas the Qwen-7B CoT model demonstrated more balanced class performance (accuracy=82.7%).

**Conclusions:**

Supervised fine-tuning of LLMs with CoT offers an effective approach for automated PHD detection within unstructured data in the electronic medical record. While CoT-enhanced models demonstrated improved internal performance and more balanced classification, they did not consistently achieve higher accuracy in external validation, highlighting trade-offs between accuracy and class balance. These findings highlight the promise of LLM-based approaches for clinical text phenotyping while underscoring the need for larger, multicenter validation and careful calibration for real-world deployment. Continued validation and integration into the electronic medical record are essential for real-world, artificial intelligence–driven clinical decision support.

## Introduction

Congenital heart defects (CHDs) are the most prevalent type of birth defect, affecting approximately 1% of live births globally [1,2]. These defects encompass a wide range of structural abnormalities of the heart that arise during fetal development [3]. Some simple abnormalities may have virtually no clinical consequences, while others may have a broad impact on clinical care [2,4,5]. Given the complexity and lifelong nature of CHDs, accurate and timely identification of affected individuals within health care systems is crucial for ensuring optimal patient outcomes [6,7]. Some patients also have acquired pediatric heart disease (PHD) that is noncongenital, and these disorders present variably as well.

The detection of PHD, including CHD, within the electronic health record (EHR) creates a significant challenge due to the heterogeneity of data sources and the variability in clinical documentation practices [8,9]. Traditional methods of identifying patients with PHD often rely on problem list diagnoses or discrete data elements, such as *International Classification of Diseases, Ninth Revision* (ICD-9) and *International Classification of Diseases, Tenth Revision* (ICD-10) codes, structured fields, and smart data elements (SDEs) [8,10-15]. However, these methods may not capture the full spectrum of PHD, particularly in cases where diagnoses are not explicitly documented or are recorded in unstructured formats, such as narrative echocardiogram reports [16,17]. Furthermore, discrete documentation within EHRs is often inadequate to determine the clinical significance of a given diagnosis.

Echocardiography serves as the primary diagnostic tool for PHD, providing detailed and real-time assessments of cardiac morphology and function [18,19]. However, echocardiogram reports are usually unstructured narratives, making automated identification of PHD challenging within EHR systems. A robust computational approach for extracting and interpreting echocardiogram reports would improve clinical decision support, risk stratification, and patient management in PHD care [20,21]. Furthermore, many echocardiogram findings may be abnormal but are not clinically relevant in certain patient-care settings. Thus, there is an additional important task of differentiating between clinically significant echocardiogram abnormalities and nonclinically significant diagnoses.

Advances in health informatics have opened new avenues for improving the detection of patients with PHD in the EHR [22]. Despite these advances, existing approaches for identifying PHD from EHRs remain limited. Traditional methods relying on structured data elements such as *International Classification of Diseases* (ICD) codes and SDEs often demonstrate incomplete capture and variable accuracy, particularly when clinical significance must be inferred rather than explicitly coded [8-11,13]. Natural language processing (NLP) enables the extraction of relevant information from unstructured text, such as echocardiogram reports, by analyzing the language and context used in clinical narratives [23,24]. However, these classical rule-based NLP approaches are limited by rigid rules and the need for manual feature engineering, which hinders their ability to generalize across diverse reporting styles to capture the full complexity of narrative clinical text [23,24].

More recently, large language models (LLMs), such as the LLaMA-3.2-3B model, have introduced new possibilities for advanced NLP applications in health informatics [25]. While LLMs improve information extraction, they may still struggle with tasks that require nuanced clinical reasoning, such as distinguishing clinically significant from incidental findings [25]. In PHD, this distinction often depends on integrating multiple findings, interpreting clinical context, and applying domain-specific judgment, which may not be consistently achieved through standard prompting [26,27].

These limitations highlight a critical gap: current computational approaches are not specifically designed to extract the structured clinical reasoning from unstructured clinical text required to determine the significance of echocardiographic findings. As a result, there remains a need for approaches that both align model behavior with expert-labeled clinical decision-making and guide the model through interpretable reasoning processes.

Supervised fine-tuning (SFT) of LLMs enhances domain-specific performance by training models on specialized datasets, adjusting weights to mimic gold standard responses using example prompts and a loss function, and enabling accurate extraction of clinically relevant findings [26,27]. Complementarily, chain-of-thought (CoT) reasoning has emerged as an effective technique for improving the interpretability and logical consistency of LLMs through intermediate reasoning steps, particularly in complex medical classification tasks [28,29]. However, the optimal integration of CoT into fine-tuned models for PHD detection, and other real-world medical applications, remains largely unexplored [30]. Therefore, understanding whether integrating CoT into fine-tuned LLMs can improve PHD classification performance or alter model behavior in clinically meaningful ways is an important open question.

This study investigates the effectiveness of SFT of LLMs with CoT reasoning for identifying PHD within unstructured echocardiogram reports and classifying whether the PHD is clinically significant by leveraging the high-contextual understanding of LLMs. By leveraging the contextual reasoning capabilities of LLMs, this approach can overcome limitations of structured data, capturing a broader range of PHD characteristics and subtle clinical nuances that are often documented in narrative form [31,32].

The significance of this research lies in its potential to improve the quality of care for patients with PHD. Accurate stratification of PHD and determining clinical significance in the context of a given specialty enable health care providers to deliver timely and appropriate interventions, anticipate clinical needs, reduce the risk of complications, and enhance overall coordination of care [33-35]. Furthermore, the insights gleaned from this study could inform the development of similar algorithms for other complex medical conditions, ultimately contributing to the advancement of precision medicine and personalized health care [36]. By harnessing the power of LLMs, this study aims to develop a robust and reliable algorithm for PHD identification, paving the way for enhanced clinical decision support and optimal patient care [23,24].

In this study, we hypothesize that SFT of LLMs, combined with CoT-generated reasoning from DeepSeek R1, can significantly enhance classification accuracy and consistency. By leveraging these approaches, we aim to establish a scalable and clinically applicable framework for PHD detection, ultimately supporting better clinical decision-making and improving patient outcomes.

## Methods

### Study Design and Population

This study aimed to develop and evaluate an algorithm for detecting PHD using unstructured echocardiogram reports. A cohort of 210,275 patients with diverse demographic and clinical characteristics was gathered from a large health care system’s EHR database. The cohort was any patient younger than 18 years of age who had anesthesia between April 1, 2014, and July 6, 2021. Among these patients, 35,585 echocardiogram encounters were available. Out of the total, 9749 reports included both ICD-10 codes and SDEs from their associated anesthesia and procedural encounter, as summarized in Tables S1 and S2 in Multimedia Appendix 1. Additionally, 712 encounters were adjudicated by 2 pediatric cardiac anesthesiologists (MCF and HDK) using a randomly stratified method. Of these, 506 (71.1%) were classified as clinically significant PHD, while 206 (28.9%) were deemed not clinically significant.

To ensure high-quality class labels, expert physician adjudication was conducted through a detailed, independent evaluation of each echocardiogram report to determine the presence of clinically significant PHD. Clinical significance was based on the viewpoint of a pediatric cardiac anesthesiologist. Reports were labeled as negative if they did not meet the defined criteria or were adjudicated as nonsignificant by the experts. The adjudication process was blinded, meaning that clinicians were not able to view each other’s assessments. For the 712 adjudicated cases, any instances of disagreement between clinicians were re-evaluated through a readjudication process, involving a third pediatric cardiac anesthesiologist (JBL), until a consensus was reached.

To assess external generalizability, an independent validation cohort of 113 Duke pediatric patients, each with a single echocardiogram report, was included. These reports were adjudicated by the same 2 pediatric cardiac anesthesiologists (MCF and HDK) using the same blinded process applied to the internal dataset. Of the 113 cases, 64 (56.6%) were classified as clinically significant PHD, while 49 (43.4%) were adjudicated as not clinically significant. This dataset allowed us to test whether the models trained internally could maintain accuracy across institutions.

### Data Sources and Elements

Unstructured echocardiogram reports contain detailed narrative descriptions of cardiac anatomy and function, providing valuable clinical information. However, the lack of standardized formatting and verbiage poses challenges for automated data extraction. MedSpaCy, a clinical NLP library built on spaCy and designed for processing clinical text, was used to extract structured information such as PHD history and current disease status from echocardiogram reports [37]. It provides rule-based and machine learning pipelines tailored for health care text, supporting tasks such as entity recognition, section segmentation, and concept mapping [37]. In this study, MedSpaCy outputs are used as a rule-based NLP baseline for comparison with LLM-based approaches, particularly in identifying PHD history and clinically significant PHD from echocardiogram reports. To address this, we used LLMs for NLP, enabling the interpretation of free-text reports. The process involved tokenization to break down text into meaningful components, followed by model-driven inference to extract and classify PHD phenotypes. By leveraging LLMs, we transformed unstructured narratives into structured insights, facilitating the development of an automated PHD detection algorithm.

### Data Processing and Analysis

The dataset included 9749 echocardiogram encounters, from which 712 encounters were adjudicated by 2 pediatric cardiac anesthesiologists using a randomly stratified approach. The adjudicated dataset (n=712) was partitioned into a training subset (71/712, 9.9%) and a held-out evaluation subset (641/712, 90%). This proportion was selected to balance model performance and computational efficiency, as preliminary experiments demonstrated that performance improved substantially at lower training proportions but plateaued beyond 10% across accuracy, sensitivity, and specificity (Figure S1 in Multimedia Appendix 1). Using a limited training subset also allowed us to maximize the size of the held-out evaluation set, thereby reducing the risk of overfitting and providing a more robust estimate of model performance. Splitting was performed at the patient level to ensure that all reports from a given patient were contained entirely within a single subset, thereby preventing overlap between training and evaluation data. Each echocardiogram report included in the adjudicated dataset corresponds to a unique patient encounter, and no patient contributed data to both the training and evaluation sets.

The fine-tuning subset was used exclusively for supervised model training, and these samples were excluded from all evaluation metrics to prevent data leakage. Only adjudicated reports were used for SFT, and no nonadjudicated data were included in the training pipeline. Model performance was assessed on the held-out evaluation subset using the physician-adjudicated labels as the reference standard. For external validation, the same fine-tuned models trained on the internal Children’s Healthcare of Atlanta (CHOA) dataset were directly evaluated on the Duke dataset (n=113) without additional fine-tuning. The external Duke dataset was entirely independent of the internal CHOA dataset, with no shared patients, reports, or overlap in data sources, ensuring a fully independent evaluation of model generalizability.

To assess robustness and account for variability in model inference, each model configuration was evaluated across 10 independent runs with identical data splits and the same sampling conditions (eg, temperature). Performance metrics were averaged across runs and reported with 95% CIs (Tables S4 and S5 and Figures S5 and S7 in Multimedia Appendix 1).

Several experimental configurations were tested, including temperature tuning to control response variability, where lower values generated deterministic outputs, while higher values allowed for the exploration of rarer PHD descriptors [38,39]. In addition, we explored zero-shot, one-shot, and few-shot learning settings to determine the model’s ability to generalize with limited labeled data.

### Model Development and Fine-Tuning

We developed an algorithm to classify PHD by analyzing unstructured echocardiogram reports using open-source fine-tuning of LLMs for high-context NLP tasks [40]. The models used included LLaMA-3.2-3B (Meta) [40,41], Qwen-2.5-3B, and Qwen-2.5-7B (Alibaba) [42]. Including both 3B and 7B models allowed us to examine scaling effects across model families, since Qwen’s smallest publicly available open-source model was 3B, making exact size-matching with LLaMA difficult. This model set enabled evaluation of whether larger Qwen models offered consistent advantages over size-matched counterparts.

**Figure 1.**
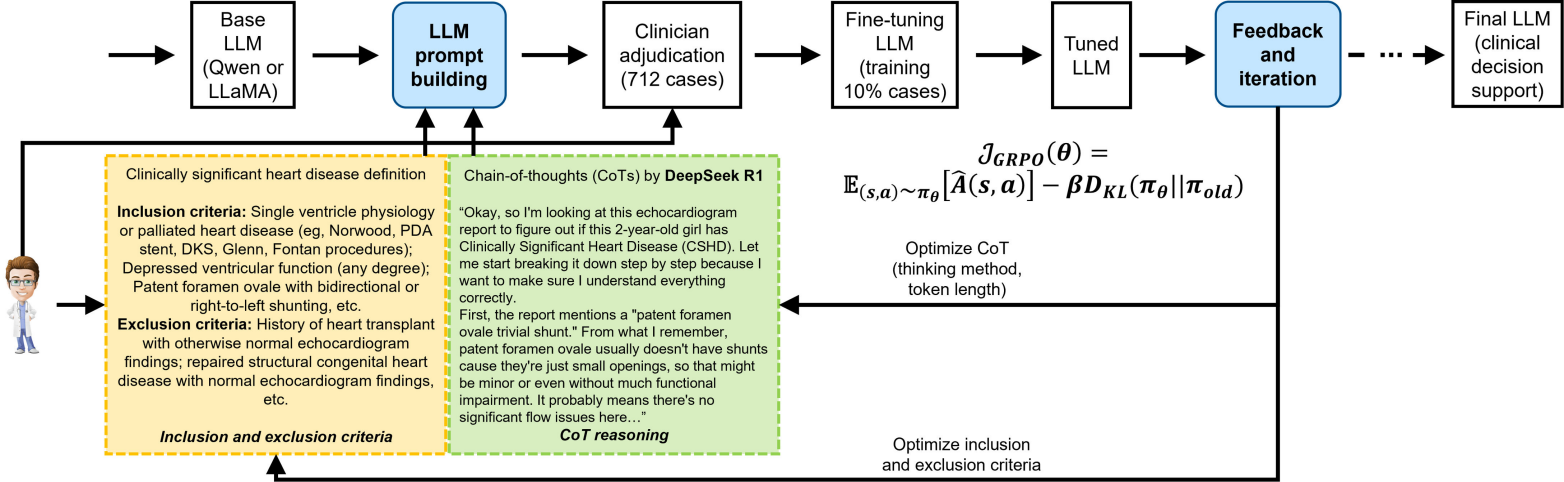
Our framework: an iterative approach of LLM prompt engineering and model fine-tuning. A schematic depicting the iterative prompt engineering and fine-tuning workflow for clinically significant pediatric heart disease classification using LLMs. The figure illustrates inclusion and exclusion criteria, chain-of-thought reasoning via DeepSeek R1, and the fine-tuning process with Qwen and LLaMA to enhance model performance. DKS: Damus-Kaye-Stansel procedure; LLM: large language model; PDA: patent ductus arteriosus.

Given the demonstrated success of DeepSeek R1 in improving performance through CoT reasoning, we integrated CoT-generated outputs from the DeepSeek-R1-Distill-Qwen-7B model into our framework [43]. CoT reasoning traces were incorporated as input prompt augmentations during both fine-tuning and inference, rather than being used as supervised target outputs, allowing the model to leverage structured reasoning without directly learning to reproduce the reasoning steps. The fine-tuning process involved adjusting CoT token length selectively during training and testing to assess its impact on performance. CoT generation includes repetitive CoT and overthinking CoT: repetitive CoT is produced by lowering the repetition penalty hyperparameter to generate less diverse, more repetitive tokens, while overthinking CoT prevents premature conclusions by replacing them with prompts that encourage deeper, iterative reasoning [43,44]. The framework pipeline is shown in Figure 1.

DeepSeek-R1 is a reasoning-focused language model that enhances its capabilities through reinforcement learning, specifically using a method called Group Relative Policy Optimization [43]. Group Relative Policy Optimization optimizes the policy model *π*_*θ*_ by maximizing the following objective function shown in Figure 1, where state (*s*) is the initial prompt or problem statement presented to the model, action (*a*) is the model’s generated response or reasoning step, policy (*π*) is the strategy the model uses to generate responses based on given prompts, reward (*r*) is a scalar value assessing the quality or correctness of the model’s response, *Â(s,a)* is the advantage function, indicating how much better an action *a* is compared to the average action in state *s*, *D*_*KL*_ is the Kullback-Leibler divergence, measuring the difference between the new policy *π*_*θ*_ and the old policy *π*_*old*_, and *β* is the coefficient controlling the importance of the divergence term, balancing policy improvement and stability.

Additionally, PHD inclusion and exclusion criteria (Textbox 1) were used during prompt building to narrow the definition for PHD; an example of the final prompt for PHD history and clinically significant PHD is provided in Multimedia Appendix 2.

Textbox 1. **Inclusion and exclusion criteria for clinically significant pediatric heart disease (PHD).**

**Inclusion criteria (significant PHD)**

- Single ventricle physiology or palliated heart disease (eg, Norwood, patent ductus arteriosus stent, Damus-Kaye-Stansel procedure, Glenn, and Fontan procedures)
- Depressed ventricular function (any degree)
- Patent foramen ovale with bidirectional or right-to-left shunting
- Pulmonary hypertension (eg, right ventricular systolic pressure or tricuspid regurgitation gradient >25 mm Hg, septal flattening, and right ventricular hypertension)
- Hypertrophic obstructive cardiomyopathy (with or without obstruction)
- Vascular stent with a stenosis gradient present
- Mechanical circulatory support
- Systemic or pulmonary venous stenosis with a gradient
- Tetralogy of Fallot
- Double outlet right ventricle
- Double inlet left ventricle
- Ventricular septal defect
- Atrial septal defect
- Atrioventricular septal defect or canal
- Transposition of the great arteries
- Hypoplastic left heart
- Ebstein anomaly
- Pulmonary atresia
- William syndrome
- Tricuspid atresia
- Patent ductus arteriosus
- Coarctation of the aorta
- Anomalous pulmonary venous return (partial or total)
- Truncus arteriosus
- Moderate to severe valvular stenosis (any valve)
- Moderate to severe valvular regurgitation or insufficiency (any valve)
- Cardiac tumors

**Exclusion criteria (no or nonsignificant PHD)**

- History of heart transplant with otherwise normal echocardiogram findings
- Repaired structural congenital heart disease with normal echocardiogram findings
- Right ventricle to pulmonary artery conduit without stenosis or regurgitation
- Repaired atrioventricular canal defects without valve stenosis or regurgitation
- Trace or mild valve regurgitation (any valve)
- Bicuspid aortic valve without stenosis or regurgitation
- Mild pericardial effusion
- Patent foramen ovale with left-to-right shunting

### Model Benchmarking

To comprehensively evaluate different fine-tuning strategies, we implemented multiple learning paradigms, including zero-shot learning with LLaMA-3.2-3B, where no labeled data were used, and few-shot learning with LLaMA-3.2-3B, which incorporated a small, labeled dataset to enhance model performance. Additionally, we used low-rank adaptation (LoRA) fine-tuning [45], a parameter-efficient technique that adapts LLMs for PHD classification by introducing trainable low-rank matrices into the attention layers while keeping the base model weights frozen, thereby reducing computational costs. The LoRA fine-tuning process was explored through various configurations (Table S3 in Multimedia Appendix 1), including LLaMA-3.2-3B, Qwen-2.5-3B, and Qwen-2.5-7B with and without CoT applied exclusively during training, and both models with varying token lengths of overthinking or repetitive CoT applied across all data.

To improve reproducibility, additional details of the fine-tuning and inference process are provided. All models were fine-tuned using LoRA with rank (r)=16, scaling factor (α)=16, and 4-bit quantization. The learning rate was set to 2×10^–5^ with a cosine decay schedule, and models were trained for 3 epochs with a batch size of 2 and gradient accumulation steps of 8. During inference, decoding was performed using a temperature of 0.2 to promote stable and consistent outputs, with top-p sampling set to 0.9. Maximum generation length was set according to the input configuration, particularly for CoT-enhanced prompts with extended token lengths. All models were evaluated under identical decoding conditions to ensure fair comparison across configurations. Unless otherwise specified, default tokenizer settings provided by each model architecture were used. These configurations were kept consistent across all experiments to isolate the effects of model architecture and CoT integration.

All experiments were conducted on a single NVIDIA L40S graphics processing unit with 48 GB of video RAM, an Intel Xeon Platinum 8358 central processing unit, and 32 GB of allocated system memory. Models were executed using Compute Unified Device Architecture–enabled PyTorch in a Linux-based high-performance computing environment.

CoT reasoning traces were generated using a separate reasoning model (DeepSeek-R1-Distill-Qwen-7B), which contains 5.34 billion parameters and required approximately 7.84 GB model memory and ~17 GB GPU allocation during inference.

The total end-to-end computational time for the best-performing configuration (Qwen-2.5-7B-10k-overthink-CoT) was approximately 11.2 hours, with the majority of time attributed to CoT generation rather than model fine-tuning.

Incorporating Qwen-2.5-3B allowed us to include a size-matched comparator to LLaMA-3B, while Qwen-2.5-7B was evaluated to assess scaling effects within the Qwen family. To assess generalizability, the same fine-tuned models trained on the internal CHOA dataset were directly evaluated on the Duke external dataset (n=113) without additional fine-tuning. Each configuration was optimized to improve PHD detection from narrative echocardiogram reports and to evaluate the impact of CoT reasoning and CoT length on model performance.

### Statistical Analysis

The performance of the PHD detection algorithm was evaluated using standard metrics, including accuracy, positive predictive value (PPV), negative predictive value (NPV), sensitivity, specificity, and *F*_1_-score. These metrics were calculated by comparing the algorithm’s output to the adjudicated gold standard. To ensure robustness and evaluate model reliability, each model configuration was evaluated across 10 independent runs, and the performance metrics were averaged.

Along with these measures, the *F*_1_-score served as an important metric for this comparison, as it provides a harmonic mean of PPV and sensitivity. This is particularly critical in the context of PHD detection, as it ensures that the model effectively balances the need to capture all positive cases with the necessity of minimizing false positives in complex clinical reports.

### Ethical Considerations

The study was approved by the CHOA Institutional Review Board (STUDY00001114) and the Duke University Health System Institutional Review Board with a waiver of informed consent (Pro00116862).

## Results

Table 1 summarizes the demographic characteristics and PHD-related classifications for the CHOA dataset (n=9749), including structured data elements (SDE), ICD-10 codes, rule-based NLP outputs (MedSpaCy), and LLM-derived classifications. Overall, 53.1% (5178/9749) of patients were identified as having PHD by ICD-10 codes and 84.9% (8279/9749) by SDEs. MedSpaCy classified 95.7% (9329/9749) of reports as clinically significant PHD, whereas LLM-based approaches classified 40.2% (3915/9749) of reports as clinically significant. This comparison with MedSpaCy highlights notable differences relative to LLM-based classification, which suggest that rule-based methods may overidentify clinically significant PHD due to limited ability to incorporate contextual clinical reasoning.

**Table 1.** Descriptive patient characteristics of Children’s Healthcare of Atlanta dataset.

	All (n=9749)	Cardio SDE^a^ (n=8279)	ICD-10^b^ (n=5178)	MedSpaCy^c^		LLM^d^	
				PHD^e^ history (n=4462)	Current PHD (n=9329)	PHD history (n=4706)	Current PHD (n=3915)
Age, n (%)							
<30 days	2246 (23)	2038 (24.6)	1597 (30.8)	761 (17.1)	2147 (23)	1297 (27.6)	1077 (27.5)
30 days-1 year	2248 (23.1)	2046 (24.7)	1561 (30.1)	1189 (26.2)	2165 (23.2)	1341 (28.5)	1134 (29)
1‐3 years	951 (9.8)	773 (9.3)	428 (8.3)	435 (9.7)	901 (9.7)	397 (8.4)	332 (8.5)
3‐6 years	1081 (11.1)	883 (10.7)	548 (10.6)	574 (12.9)	1033 (11.1)	509 (10.8)	427 (10.9)
6‐12 years	1550 (15.9)	1257 (15.2)	622 (12)	782 (17.5)	1484 (15.9)	626 (13.3)	520 (13.3)
12‐18 years	1672 (17.2)	1281 (15.5)	422 (8.1)	721 (16.2)	1598 (17.1)	536 (11.4)	425 (10.9)
18+ years	1 (0)	1 (0)	0 (0)	0 (0)	1 (0)	0 (0)	0 (0)
Sex, n (%)							
Male	5454 (55.9)	4621 (55.8)	2864 (55.3)	2434 (54.5)	5211 (55.9)	2593 (55.1)	2133 (54.5)
Female	4295 (44.1)	3658 (44.2)	2314 (44.7)	2028 (45.5)	4118 (44.1)	2113 (44.9)	1782 (45.5)
Race, n (%)							
Asian	350 (3.6)	294 (3.6)	183 (3.5)	158 (3.5)	338 (3.6)	155 (3.3)	135 (3.4)
Black or African American	3700 (38)	3099 (37.4)	1863 (36)	1653 (37)	3538 (37.9)	1726 (36.7)	1407 (35.9)
White	4676 (48)	4030 (48.7)	2625 (50.7)	2250 (50.4)	4466 (47.9)	2363 (50.2)	2017 (51.5)
Other or unknown	1023 (10.5)	856 (10.3)	507 (9.8)	401 (9)	987 (10.6)	462 (9.8)	356 (9.1)
Ethnicity, n (%)							
Hispanic	1337 (13.7)	1118 (13.5)	685 (13.2)	568 (12.7)	1271 (13.6)	610 (13)	517 (13.2)
Non-Hispanic	8396 (86.1)	7151 (86.4)	4489 (86.7)	3890 (87.2)	8043 (86.2)	4093 (87)	3396 (86.7)
Other or unknown	16 (0.2)	10 (0.1)	4 (0.1)	4 (0.1)	15 (0.2)	3 (0.1)	2 (0.1)
Heart disease, n (%)							
PHD by ICD-10	5178 (53.1)	5073 (61.3)	5178 (100)	3224 (72.3)	4955 (53.1)	3651 (77.6)	3133 (80)
PHD by SDE	8279 (84.9)	8279 (100)	5073 (98)	4143 (92.9)	7958 (85.3)	4515 (95.9)	3743 (95.6)
PHD history by MedSpaCy from echocardiogram reports	4462 (45.8)	4143 (50)	3224 (62.3)	4462 (100)	4043 (43.3)	2822 (60)	2409 (61.5)
Clinically significant PHD by MedSpaCy from echocardiogram reports	9329 (95.7)	7958 (96.1)	4955 (95.7)	4043 (90.6)	9329 (100)	4494 (95.5)	3730 (95.3)
PHD history by LLM from echocardiogram reports	4706 (48.3)	4515 (54.5)	3651 (70.5)	2822 (63.2)	4494 (48.2)	4706 (100)	3824 (97.7)
Clinically significant PHD by LLM from echocardiogram reports	3915 (40.2)	3742 (45.2)	3133 (60.5)	2409 (54)	3730 (40)	3824 (81.3)	3915 (100)
Length of echocardiogram report, median (IQR)							
Words	168.0 (109.0-772.0)	169.0 (110.0-798.0)	172.0 (112.0-814.0)	178.0 (111.2-797.0)	167.0 (109.0-770.0)	613.0 (136.0-945.0)	622.0 (138.0-957.0)
Tokens	215.0 (141.0-985.0)	216.0 (141.0-1019.0)	217.5 (143.0-1045.0)	223.0 (142.0-1014.0)	213.0 (140.0-982.0)	793.0 (172.0-1195.0)	803.0 (177.0-1210.5)
Living status, n (%)							
Alive	8688 (89.1)	7385 (89.2)	4676 (90.3)	4057 (90.9)	8300 (89)	4222 (89.7)	3505 (89.5)
Deceased	1061 (10.9)	894 (10.8)	502 (9.7)	405 (9.1)	1029 (11)	484 (10.3)	410 (10.5)
Financial class, n (%)							
Medicaid	5863 (60.1)	4954 (59.8)	3089 (59.7)	2646 (59.3)	5615 (60.2)	2801 (59.5)	2327 (59.4)
Managed care	3374 (34.6)	2902 (35.1)	1835 (35.4)	1579 (35.4)	3234 (34.7)	1660 (35.3)	1385 (35.4)
Tricare	266 (2.7)	231 (2.8)	165 (3.2)	135 (3)	256 (2.7)	145 (3.1)	125 (3.2)
Medicare	31 (0.3)	30 (0.4)	3 (0.1)	13 (0.3)	31 (0.3)	7 (0.1)	5 (0.1)
Other	215 (2.2)	162 (2)	86 (1.7)	89 (2)	193 (2.1)	93 (2)	73 (1.9)

a SDE: smart data element.

b ICD-10: International Classification of Diseases, Tenth Revision.

c MedSpaCy: medical natural language processing using spaCy.

d LLM: large language model.

e PHD: pediatric heart disease.

**Figure 2.**
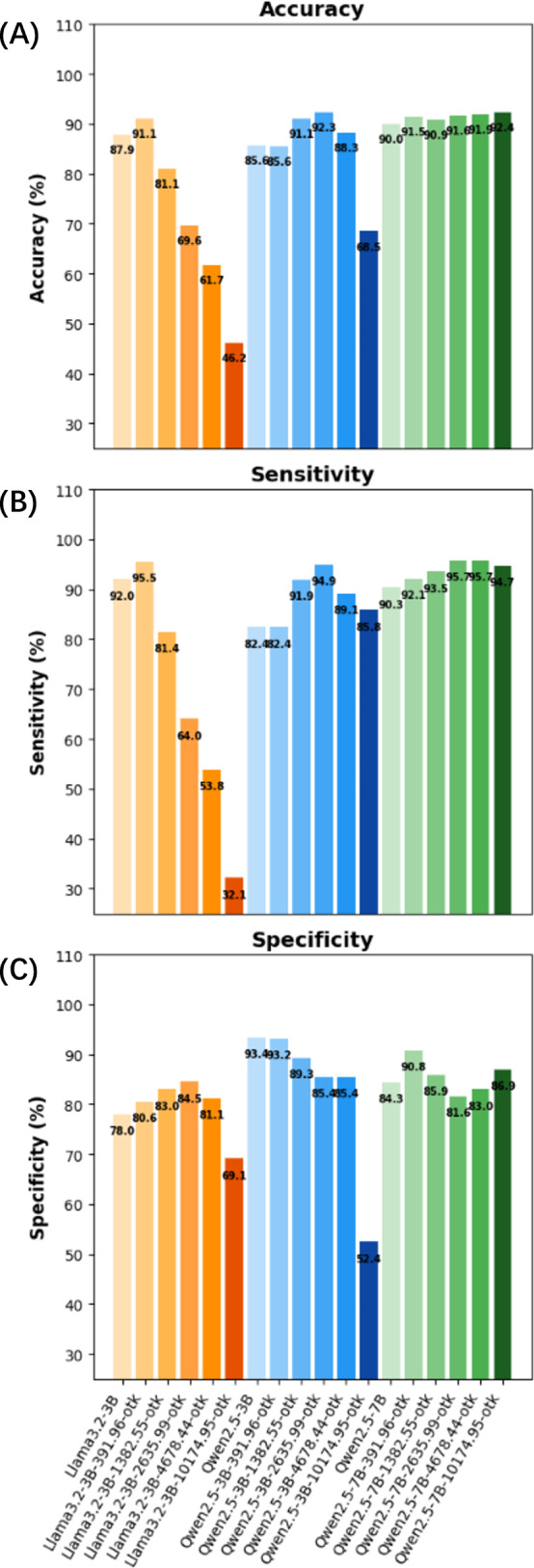
Comparison of CHOA overthinking CoT LLM model performance in PHD detection: accuracy, sensitivity, and specificity. This figure compares the performance of different LLMs for PHD detection using unstructured echocardiogram reports with overthinking CoT on the entire CHOA dataset. Orange, blue, and green bars denote LLaMA-3B, Qwen-2.5-3B, and Qwen-2.5-7B model families, respectively, with darker shades indicating increasing CoT token length. The results are divided into 3 panels: (A) overall accuracy, measuring the correctness of predictions for both patients with clinically significant PHD and not pediatric significant cases; (B) sensitivity for patients with clinically significant PHD; and (C) specificity for nonsignificant pediatric patients. Each of the 3 subplots shows bar charts for different LLMs, comparing their respective accuracies on each metric. The x axis lists the various models, and the y axis indicates percentage accuracy. Each bar’s height reflects how effectively the corresponding model performs in that category. CHOA: Children’s Healthcare of Atlanta; CoT: chain-of-thought; LLM: large language model; otk: overthinking CoT; PHD: pediatric heart disease.

Figure 2 presents a comparative analysis of different SFT models for PHD classification using overthinking CoT. All reported performance metrics represent the mean across 10 independent runs, with variability summarized using 95% CIs. The figure highlights distinct performance trends across varying training paradigms, demonstrating the impact of overthinking CoT, the length of CoT, and the base LLM selection, on model accuracy and overall classification performance. Among these models, the Qwen-2.5-7B-R1-overthink-CoT-10k-token model emerged as the top-performing approach, achieving the highest total accuracy of 92.4%, along with 94.7% sensitivity and 86.9% specificity (Figure S6 in Multimedia Appendix 1). Accuracy increased with increasing CoT token length for Qwen-7B models, reaching a peak at 10,000 tokens.

Among the tested models, Qwen-based models consistently outperformed LLaMA-based models across all accuracy metrics. Moreover, Qwen-2.5-7B models surpassed their Qwen-2.5-3B counterparts, with the largest performance gap observed in both accuracy and sensitivity. Specifically, in 4 of 6 (66.7%) model comparisons, the 7B models exceeded the 3B models in total accuracy, and in all 6 (100%) comparisons, the 7B models exceeded the 3B models in sensitivity. While both model sizes benefited from overthinking CoT, the Qwen-2.5-7B architecture capitalized more strongly, suggesting that larger models are better able to exploit long-form reasoning. Overall, these results suggest that Qwen’s architecture, particularly at a larger scale, may be better suited for medical text processing in PHD classification.

**Figure 3.**
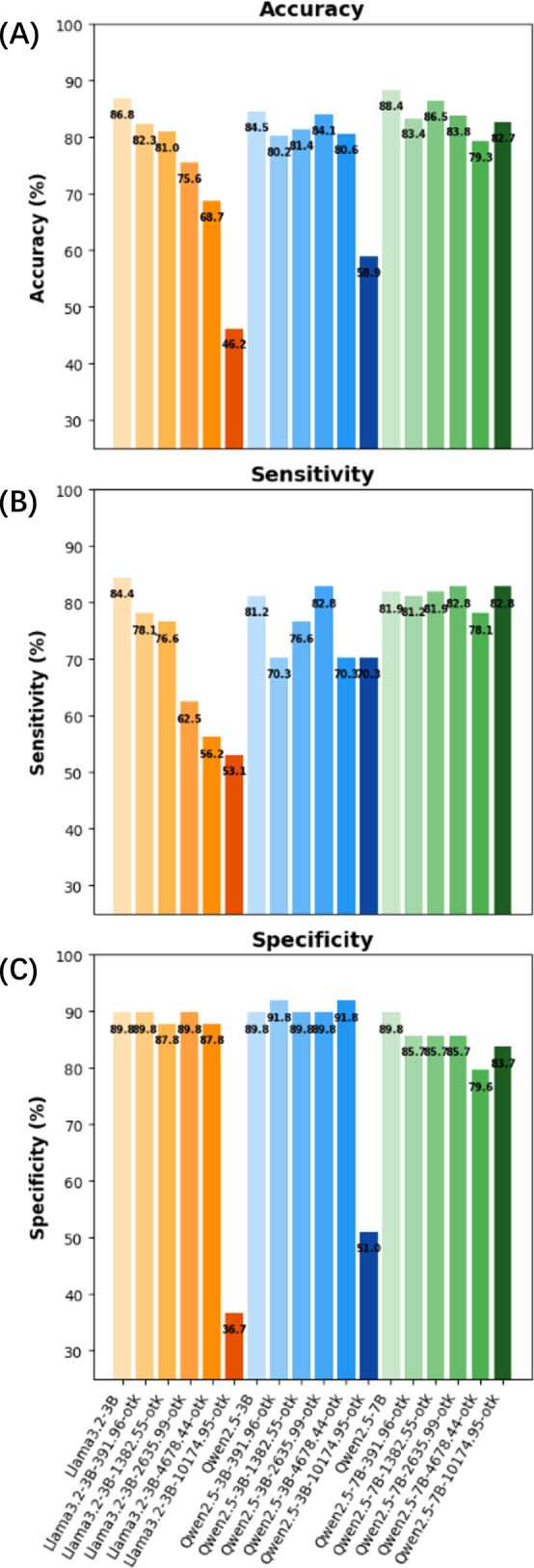
External validation of overthinking CoT LLM model performance in PHD detection: accuracy, sensitivity, and specificity. This figure compares the performance of different LLMs for PHD detection using unstructured echocardiogram reports with overthinking CoT on an independent external dataset of 113 Duke pediatric patients. Orange, blue, and green bars denote LLaMA-3B, Qwen-2.5-3B, and Qwen-2.5-7B model families, respectively, with darker shades indicating increasing CoT token length. The results are divided into 3 panels: (A) overall accuracy, measuring the correctness of predictions for both patients with clinically significant PHD and not pediatric significant cases; (B) sensitivity for patients with clinically significant PHD; and (C) specificity for nonsignificant pediatric patients. Each of the 3 subplots shows bar charts for different LLMs, comparing their respective accuracies on each metric. Each subplot displays bar charts of model accuracy on the external dataset, with the x axis listing the models and the y axis showing accuracy percentages. The evaluation highlights model generalizability beyond the training dataset. CoT: chain-of-thought; LLM: large language model; otk: overthinking CoT; PHD: pediatric heart disease.

Figure 3 extends the evaluation to an external dataset of 113 Duke pediatric patients (Table 2), providing an independent test of model generalizability. In external validation, models without CoT achieved higher overall accuracy, whereas CoT-enhanced models demonstrated more balanced sensitivity and specificity. Although the Qwen-2.5-7B-Instruct model achieved the highest total accuracy (88.4%), the Qwen-2.5-7B-R1-overthink-CoT-10k-token model again demonstrated the most balanced positive and negative class performance, achieving 82.7% accuracy, 82.8% sensitivity, and 83.7% specificity. This balance suggests that the overthinking CoT approach improves robustness across both clinically significant and nonsignificant cases, rather than optimizing for only 1 class.

**Table 2.** Descriptive patient characteristics of the external Duke dataset.

Characteristics	All (n=113)
Age, n (%)	
<30 days	6 (5.3)
30 days-1 year	21 (18.6)
1‐3 years	12 (10.6)
3‐6 years	18 (15.9)
6‐12 years	24 (21.2)
12‐18 years	27 (23.4)
18+ years	5 (4.4)
Sex, n (%)	
Male	60 (53.1)
Female	53 (46.9)
Race, n (%)	
Asian	1 (0.9)
Black or African American	32 (28.3)
White	65 (57.5)
Other or unknown	15 (13.3)
Ethnicity, n (%)	
Hispanic	9 (8)
Non-Hispanic	97 (85.8)
Other or unknown	7 (6.2)
Length of echocardiogram report, median (IQR)	
Words	391.0 (348.0-436.0)
Tokens	453.0 (402.0-506.0)
Financial class, n (%)	
Medicaid	52 (46)
Managed care	19 (16.8)
Tricare	0 (0)
Medicare	1 (0.1)
Other	41 (36.3)

When examining the impact of overthinking token length, clear trade-offs were observed. For both LLaMA-3B and Qwen-2.5-3B models, total accuracy declined progressively as overthinking tokens increased, with a sharp drop once token counts exceeded 10,000. In contrast, the Qwen-2.5-7B architecture showed greater stability, with the R1 overthink 10,000-token model achieving the most balanced external validation performance across sensitivity and specificity.

**Figure 4.**
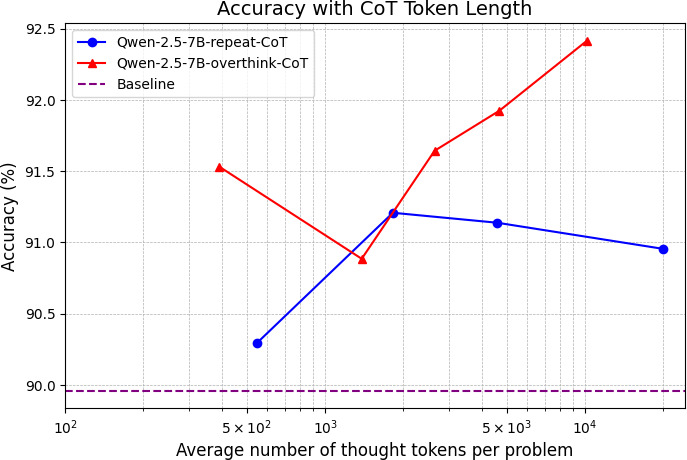
PHD detection overall accuracy with CoT token length for Qwen-2.5-7B. This figure illustrates how overall PHD detection accuracy changes as the average number of tokens in the CoT increases. The blue line with circular markers represents Qwen-2.5-7B using DeepSeek R1’s repetitive CoT, while the red line with triangular markers represents Qwen-2.5-7B using DeepSeek R1’s overthinking CoT, and the dashed purple line indicates the baseline accuracy using Qwen-2.5-7B without CoT. The x axis shows the average number of CoT tokens per echocardiogram report, and the y axis shows the corresponding accuracy in percentage. CoT: chain-of-thought; PHD: pediatric heart disease.

Figure 4 further explores the impact of CoT token length on model accuracy and highlights the trade-off between reasoning depth and computational efficiency. The analysis reveals that, as the average CoT token length increases, accuracy improves significantly for the overthink CoT approach, with Qwen-2.5-7B reaching its peak performance at 92.4% with an average of ~10,000 tokens per problem. Meanwhile, the repetitive CoT approach showed a plateau in accuracy beyond 2000 token length, suggesting diminishing returns from excessive repetition. Notably, smaller models showed performance degradation at longer token lengths, suggesting that excessive reasoning sequences may exceed their effective context use capacity. These findings emphasize that the importance of optimizing CoT strategies depends not only on token length but also on the interaction between reasoning strategy and model scale rather than simply increasing token length, and that optimal performance requires careful calibration of reasoning depth relative to computational cost.

To further characterize the computational impact of CoT reasoning, we evaluated the relationship between model performance and total inference time across different model sizes and CoT configurations (Figure S2 in Multimedia Appendix 1). As the CoT token length increased, total computation time increased substantially across all models, particularly for larger architectures. While Qwen-2.5-7B maintained stable performance with increasing computational cost, smaller models (eg, LLaMA-3B and Qwen-3B) demonstrated performance degradation at longer CoT lengths despite increased inference time. These findings highlight a clear trade-off between reasoning depth and computational efficiency, indicating that longer CoT sequences do not uniformly translate to improved performance.

**Figure 5.**
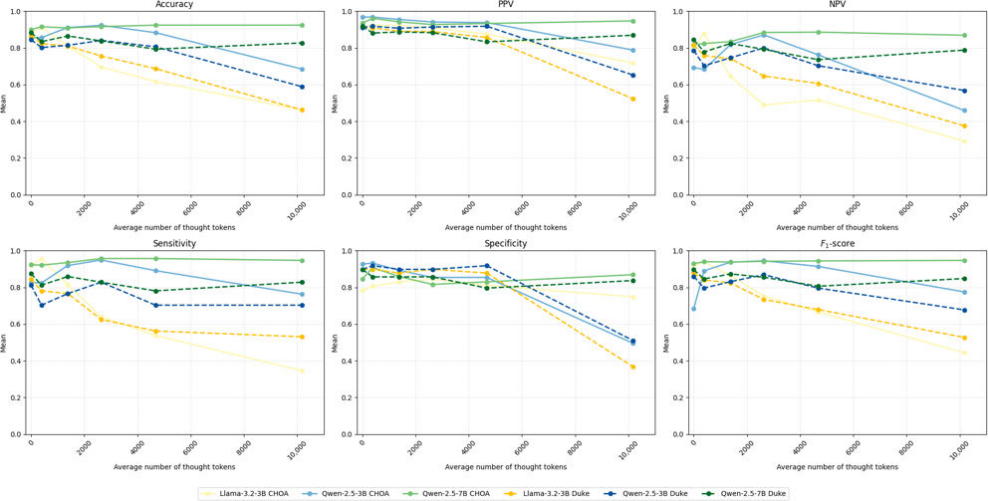
Multimetric performance of CoT-enhanced LLMs across CoT token lengths in internal (CHOA) and external (Duke) validation cohorts. This figure illustrates the relationship between reasoning depth (categorized by average CoT token length: No-CoT, 400, 1400, 2600, 4700, and 10,000 “overthink”) and model performance across multiple LLM variants. Evaluation metrics are shown for accuracy, PPV, NPV, sensitivity, specificity, and *F*_1_-score. Performance is contrasted between the internal training site (CHOA) and the external validation site (Duke) to assess model generalizability and the differential impact of extended reasoning on cross-institutional data. CHOA: Children’s Healthcare of Atlanta; CoT: chain-of-thought; LLM: large language model; NPV: negative predictive value; PPV: positive predictive value.

Figure 5 summarizes the impact of CoT incorporation and CoT token length on a comprehensive set of classification metrics, including accuracy, sensitivity, specificity, PPV, NPV, and *F*_1_-score, evaluated on both the internal CHOA dataset and the external Duke validation cohort. Across all metrics, model performance metrics were consistently higher at CHOA than at Duke, highlighting the expected performance attenuation during external validation. For CHOA, model performance generally improved with the incorporation of CoT overthinking reasoning, and model performance further improved as CoT length increased, particularly for the Qwen-2.5-7B variants. The best-performing CHOA configuration was Qwen-2.5-7B-10k-otk, achieving an *F*_1_-score of 0.95, reflecting excellent balance between PPV and sensitivity.

Conversely, the Duke validation set demonstrated a divergent trend. The introduction of CoT generally led to a performance drop compared to baseline models. The highest performance at Duke was achieved by the Qwen-2.5-7B model without CoT, yielding an *F*_1_-score of 0.90, whereas increasing CoT length tended to reduce overall accuracy. Despite this trend, the Qwen-2.5-7B-10k-otk configuration provided the most balanced performance for the Duke cohort among the overthink strategies, maintaining an *F*_1_-score of 0.85. Together, these findings demonstrate that while CoT reasoning substantially enhances internal performance, its application in external settings requires careful calibration to balance generalizability and reasoning depth.

We further evaluated model reliability by assessing the calibration of the Qwen-2.5-7B-10k-otk model across different pediatric age groups (Figure S3 in Multimedia Appendix 1). Overall, the model demonstrated good calibration across age groups, with ground truth fractions closely following the line of perfect calibration. Calibration performance was consistent across neonatal (<30 days), infant (30 days to 1 year), and childhood age strata, with only minor deviations observed at lower predicted probability ranges, particularly in smaller-sized age cohorts. These deviations were most pronounced in age groups with fewer samples, reflecting increased variability rather than systematic bias. Importantly, high predicted probability ranges (≥0.9) remained well calibrated across all age categories, supporting the reliability of model confidence estimates for clinically significant PHD across diverse pediatric age groups. To ensure the statistical integrity of the visualization, probability bins with fewer than 5 cases were excluded from the plot.

Figure S4 in Multimedia Appendix 1 shows the results of the SFT models for clinically significant PHD classification and indicates clear distinctions in performance across different learning paradigms. In terms of total accuracy, the Qwen-2.5-7B-Instruct-CoT-10%-Training model achieved the highest performance at 90.3%, closely followed by Qwen-2.5-7B-Instruct-CoT at 89.2%. This suggests that selectively applying CoT reasoning to a portion of the training data enhances model generalization. The LLaMA-3.2-3B-Instruct model without CoT reached 87.7%, highlighting its strong baseline performance, though it was surpassed by CoT-enhanced models. In contrast, zero-shot (82.1%) and few-shot learning (79.2%) exhibited the lowest accuracy, reaffirming the need for fine-tuning.

Qwen-2.5-7B-Instruct demonstrated the highest sensitivity (97.7%), excelling in correctly identifying PHD cases, while zero-shot learning also performed well at 94.7%, despite its lower overall accuracy. Few-shot learning showed a decline at 81.0%, and LLaMA-3.2-3B-Instruct-CoT (training only) had the lowest sensitivity (76.7%), indicating limited generalizability when CoT is applied only during training. For specificity, Qwen-2.5-7B-Instruct-CoT-10%-Training led with 91.6%, ensuring strong differentiation of non-PHD cases, followed by LLaMA-3.2-3B-Instruct-CoT at 89.4%. In contrast, Qwen-2.5-7B-Instruct without CoT struggled with specificity (70.6%), and the zero-shot model performed the worst at 50.4%, highlighting its difficulty in distinguishing echocardiogram findings that were not clinically significant. Few-shot learning improved over zero-shot at 75.4% but remained inferior to fine-tuned models.

Overall, these findings highlight the superiority of Qwen-2.5-7B models, particularly those incorporating CoT reasoning. Fine-tuning with Qwen-2.5-7B-R1-overthink-CoT-10k-token-10%-Training yielded the best balance of accuracy, sensitivity, and specificity, suggesting that selectively applying overthinking CoT enhances model generalization and provides a balanced classification performance. While zero-shot and few-shot learning methods showed promise in PHD classification, they were significantly less reliable in identifying non-PHD cases. The LLaMA models performed well, but they generally lagged behind the Qwen models, indicating that Qwen’s architecture may be better suited for this task.

The fine-tuned Qwen-2.5-7B model, incorporating a DeepSeek R1-generated overthinking CoT averaging 10,174.95 tokens, achieved 92.4% accuracy in PHD detection at a 0.5 threshold, demonstrating strong classification performance. The model effectively classified both PHD-positive and PHD-negative cases, with a PPV of 94.7% and an NPV of 86.9%, indicating a high proportion of true positive predictions while still maintaining reasonable precision for non-PHD cases. The model achieved 94.7% sensitivity and 86.9% specificity, demonstrating a well-balanced and reliable classification performance. The *F*_1_-score, which balances precision and recall, was 94.7%, confirming strong classification capability. The macroaveraged *F*_1_-score of 90.8% and the weighted *F*_1_-score of 92.4% further emphasize the model’s effectiveness across the dataset. All reported metrics represent the mean across 10 independent runs. Observed differences in performance should be interpreted in the context of overlapping CIs (Tables S4 and S5 in Multimedia Appendix 1), as variability across runs may influence the apparent magnitude of differences between models.

## Discussion

### Principal Findings

The results of this study demonstrate the effectiveness of SFT of LLMs in detecting clinically significant PHD from unstructured echocardiogram reports. Because CoT integration in this study involves multiple factors, including longer input sequences and reasoning traces generated from an external model, the independent contribution of CoT cannot be fully disentangled. Accordingly, the observed results reflect the combined effect of CoT-augmented prompting within this framework rather than the isolated impact of CoT alone.

Among the models tested, the fine-tuned Qwen-2.5-7B model, incorporating a DeepSeek R1-generated overthinking CoT prompt averaging 10,174.95 tokens, achieved the highest overall accuracy (92.4%), outperforming other fine-tuned models, including Qwen-2.5-3B and LLaMA-based approaches. This suggests that integrating CoT reasoning into model training contributes to improved cross-institutional performance and classification performance. Notably, the model maintained a high sensitivity (94.7%) and specificity (86.9%), demonstrating a balanced ability to correctly identify both PHD-positive and PHD-negative cases.

However, these performance gains did not consistently translate to external validation. External validation on an independent Duke pediatric dataset (n=113) confirmed the overall superiority of Qwen-based models but also highlighted important differences. The Qwen-2.5-7B-Instruct model achieved the highest total accuracy (88.4%), while the Qwen-2.5-7B-R1-overthink-CoT-10k-token model provided the most balanced performance (82.7% total, 82.8% sensitivity, and 83.7% specificity). These findings reinforce that total accuracy does not capture clinically meaningful performance, and that balancing sensitivity and specificity is critical for clinical application.

Taken together, these findings also suggest that CoT influences classification behavior by improving class balance and consistency rather than uniformly increasing accuracy, particularly in external validation settings. This distinction is important in clinical applications, where reliance on accuracy alone may obscure meaningful trade-offs between sensitivity and specificity.

Our findings regarding the performance divergence between CHOA and Duke cohorts raise a critical question in the deployment of clinical LLMs: the trade-off between institutional fine-tuning and broad generalizability. While the goal of many artificial intelligence initiatives is to create a “universal” model, our results suggest that a site-specific model, optimized for the unique linguistic nuances, reporting templates, and clinical documentation styles of a single institution, may offer superior clinical utility. At CHOA, the integration of overthinking CoT reasoning led to near-perfect *F*_1_-scores; yet, the same strategy saw diminishing returns when applied to the external Duke dataset (Figure S8 in Multimedia Appendix 1). This discrepancy suggests that instead of pursuing a generalized model that performs adequately across all institutions, the more effective path toward precision medicine may be the development of locally adapted LLMs. By prioritizing local optimization, hospitals can leverage reasoning-enhanced LLMs that are finely tuned to their specific patient populations and documentation standards, ultimately providing higher reliability for local clinicians than a one-size-fits-all solution.

### Strengths and Limitations

In addition to discrimination and accuracy metrics, we evaluated model calibration across pediatric age groups to assess the reliability of predicted probabilities in clinically relevant subpopulations (Figure S3 in Multimedia Appendix 1). Overall, the model demonstrated good calibration across neonatal, infant, and childhood age strata, with predicted probabilities closely aligning with observed outcome frequencies. Minor deviations from perfect calibration were observed at lower predicted probability ranges, particularly in smaller age cohorts, likely reflecting increased variance due to limited sample sizes rather than systematic age-related bias. Importantly, calibration remained stable at higher predicted probability ranges, supporting the clinical reliability of model confidence estimates across diverse pediatric age groups. These findings suggest that the proposed approach demonstrates promising cross-site performance but also across age strata, reinforcing its potential utility for age-agnostic clinical decision support.

One of the key findings of this study is the substantial performance improvement observed when CoT reasoning was incorporated. The Qwen-2.5-7B-Instruct-CoT model, which applied CoT reasoning across all data but used only 20 samples for training, showed a 14% increase in specificity compared to the same model without CoT, demonstrating its effectiveness in correctly identifying non-PHD cases. Even more impressively, the Qwen-2.5-7B-CoT-10%-Training model achieved a 21% gain in specificity, further validating the hypothesis that incorporating CoT with more training data not only improves classification performance but also enhances interpretability in medical NLP tasks.

A particularly notable observation was the diminished performance of both LLaMA-3.2-3B and Qwen-2.5-3B models with increasing CoT token length. Unlike the Qwen-2.5-7B models, which benefited from extended CoT reasoning, the smaller 3B model’s accuracy did not improve proportionally as CoT token length increased. Instead, performance degraded when CoT token lengths exceeded a certain threshold, 10,000 tokens. This phenomenon can be attributed to the inherent limitations of smaller LLMs in handling excessively long prompts. Models less than 3B parameters tend to struggle with processing and using long-context information efficiently [46]. As the CoT token length increases, the model becomes overwhelmed by excessive reasoning steps, leading to a decline in performance rather than an improvement. In addition, as shown in Figure S2 in Multimedia Appendix 1, increasing CoT token length is associated with substantial increases in total computation time, without consistent performance gains across all model architectures. This suggests that while CoT reasoning can improve performance, its effectiveness depends on both the model’s capacity and computational cost, highlighting a trade-off between reasoning depth and efficiency and indicating that longer CoT sequences should be applied selectively.

Furthermore, the analysis presented in Figure 4 provides additional insights into the effects of different CoT strategies. Repetitive CoT exhibited no significant improvements in classification performance beyond a token length of approximately 1800, suggesting that further repetition fails to enhance the model’s understanding of the task. In contrast, overthinking CoT demonstrated a gradual improvement in accuracy as token length increased. However, when the token length was below 2600, the accuracy for significant PHD and nonclinically significant PHD classifications became increasingly unbalanced. This imbalance suggests that while the model was refining its reasoning process, it struggled to maintain a consistent ability to distinguish PHD-significant from PHD-nonsignificant cases. Notably, after surpassing the 2600 token length threshold, the accuracy for both significant and nonsignificant cases became more balanced, reinforcing the idea that a minimum amount of reasoning is necessary to achieve optimal classification performance.

The study also highlights the limitations of zero-shot and few-shot learning models. While zero-shot learning demonstrated unexpectedly high sensitivity (94.7%), its specificity was the lowest (50.4%), indicating difficulty in differentiating non-PHD cases. This suggests that while general LLMs have some inherent knowledge of PHD-related concepts, fine-tuning is necessary to improve classification robustness and reliability. Few-shot learning improved upon zero-shot in specificity but still underperformed compared to fine-tuned models, reinforcing the importance of specialized training for medical NLP applications.

### Future Directions

These findings emphasize the need for further refinement of computational phenotyping algorithms using SFT and CoT techniques. While our approach significantly improves PHD classification, challenges remain in achieving optimal trade-offs between sensitivity and specificity, particularly across internal and external datasets. Future work should explore additional strategies for fine-tuning, such as reinforcement learning with human feedback and active learning, to further enhance model reliability. Multicenter external validation will be particularly important to confirm generalizability.

One future clinical application of this technology will be to fine-tune prompting to delineate discrete features from unstructured pediatric echocardiogram reports and output them as model elements to include in predictive models; thus, enhancing model development through automated echocardiogram feature extraction. In addition, the use of automated, clinically significant PHD detection offers a reasonable clinical decision support hook to support clinical workflows in the EHR. The results further reinforce the notion that LLMs will play a role processing unstructured electronic medical record data in a way that NLP has been unable to do. Clinicians encounter enormous volumes of data in the electronic medical record during each encounter; therefore, improving the ability of LLMs to summarize that data in support of clinical workflows is crucial.

The model is limited in its ability to be generalized to other centers because many echocardiogram reports contain historical data as well as metadata regarding a patient’s clinical course. Each of these elements can obfuscate the model’s interpretation of the echocardiogram, and these structured reports vary significantly between centers. Centers with the ability to do so may benefit from local fine-tuning of models in clinical applications. Clinical significance in this study was determined by pediatric cardiac anesthesiologists based on whether the echocardiographic finding was clinically significant in that setting, but different clinical applications will require unique modifications to the model (ie, prompting). Other clinical applications will have different interpretations of clinical significance. Although performance on the external Duke dataset was slightly lower than on the CHOA dataset, the results remained strong, demonstrating that fine-tuned LLMs, particularly those incorporating CoT reasoning, provide a highly accurate and reliable method for identifying clinically significant PHD from unstructured echocardiogram reports. Centers without the ability to create locally fine-tuned models may still benefit from clinical applications of models like the one developed in this study, but have to understand the limitations of models that have not been fine-tuned on center-specific data. Thus, the findings of this study highlight both the promise and the generalizability of this approach, while also underscoring the importance of continued multicenter and local validation. Moreover, prospective validation across diverse clinical settings, together with feedback from medical experts, will be crucial for assessing the practical utility of these models in patient care.

### Conclusions

This study demonstrates that fine-tuned LLMs, particularly those incorporating CoT reasoning, provide a highly accurate and reliable method for identifying clinically significant PHD from unstructured echocardiogram reports. The Qwen-2.5-7B-R1-overthink-CoT-10k-token model achieved the highest total accuracy (92.4%) for the CHOA dataset, while external validation on the Duke dataset demonstrated promising cross-institutional performance but requires larger multicenter validation, with the Qwen-2.5-7B-Instruct model achieving the highest total accuracy (88.4%) and the Qwen-2.5-7B-R1-overthink-CoT-10k-token model demonstrating the most balanced performance across positive and negative classes. While CoT-enhanced models demonstrated improved internal performance and more balanced classification, they did not consistently achieve higher accuracy in external validation, highlighting trade-offs between accuracy and class balance. These findings indicate that fine-tuned LLMs can provide clinically meaningful performance across both internal and external datasets, making them a promising tool for clinical decision support.

Our results highlight the advantages of integrating CoT into SFT, improving the model’s ability to distinguish clinically significant PHD from normal echocardiogram findings or reports that had nonsignificant PHD documented. The findings also emphasize that while overthinking CoT provides gradual accuracy improvements with increasing token length, performance becomes more balanced only after surpassing a certain threshold (approximately 2600 tokens). In contrast, repetitive CoT does not yield significant benefits beyond 1300 tokens, suggesting that excessive repetition does not enhance classification performance.

Additionally, the study underscores the limitations of zero-shot and few-shot learning approaches in complex medical classification tasks, emphasizing the necessity of specialized fine-tuning. Both LLaMA-3B and Qwen-2.5-3B models showed diminishing performance as CoT token length increased, with sharp declines once tokens exceeded 10,000, underscoring the challenges of long-context processing in smaller models. In contrast, the Qwen-2.5-7B models remained stable with longer reasoning chains, reinforcing the importance of model selection in CoT applications.

Future research should focus on further optimizing CoT integration, validating model performance in prospective clinical settings, and exploring ways to improve negative case classification. With continued development, LLMs fine-tuned for medical applications could play a crucial role in enhancing diagnostic accuracy, streamlining clinical workflows, and ultimately improving patient outcomes.

## Supplementary material

10.2196/90968 Multimedia Appendix 1 Supplemental tables and figures including ICD-10-CM (International Classification of Diseases, Tenth Revision, Clinical Modification), and smart data element inclusion criteria, detailed model performance metrics, computational benchmarking, calibration analyses, external validation results, and additional chain-of-thought model evaluation experiments for pediatric heart disease detection.

10.2196/90968 Multimedia Appendix 2 Prompt templates for chain-of-thought generation and model training.

## Abbreviations

CHD: congenital heart defect
CHOA: Children’s Healthcare of Atlanta
CoT: chain-of-thought
EHR: electronic health record
ICD: International Classification of Diseases
ICD-10: International Classification of Diseases, Tenth Revision
ICD-9: International Classification of Diseases, Ninth Revision
LLM: large language model
LoRA: low-rank adaptation
NLP: natural language processing
NPV: negative predictive value
PHD: pediatric heart disease
PPV: positive predictive value
SDE: smart data element
SFT: supervised fine-tuning
