# Supplementary material for: Supervised Fine-Tuning of Large Language Models With Chain-of-Thought Reasoning for Pediatric Heart Disease Detection in Unstructured Echocardiogram Reports: Algorithm Development and Validation
Source: JMIR Form Res. 2026 Jun 8;10:e90968. doi: 10.2196/90968 (PMC13245642; doi:10.2196/90968)
Supplement: Multimedia Appendix 1 [file formative-v10-e90968-s001.docx]

**Table S1. ICD-10-CM PHD Code Inclusion Criteria.**

| **Code Definition** | **ICD-10-CM** |
| --- | --- |
| Hypoplastic Left Heart Syndrome (HLHS) | Q23.4 |
| Tricuspid Atresia, stenosis or absence | Q22.4 |
| Hypoplastic right heart syndrome | Q22.6 |
| Single ventricle, cor triloculare, double inlet left ventricle | Q20.4 |
| Pulmonary valve atresia or absence | Q22.0 |
| Truncus Arteriosus, Common Truncus | Q20.0 |
| Double outlet Right ventricle (DORV) | Q20.1 |
| Double outlet Left ventricle (DOLV) | Q20.2 |
| Tetralogy of Fallot | Q21.3 |
| Transposition of the Great arteries (TGA), Complete TGA, dextro-TGA, TGA not otherwise specified, classical TGA | Q20.3 |
| Congenital Corrected transposition of great arteries (CCTGA), levo-TGA | Q20.5 |
| Endocardial cushion defect; Atrioventricular septal defect; Complete atrioventricular canal defect (CAVCD) Endocardial cushion defect unspecified Endocardial cushion defect, other | Q21.2 |
| Interrupted aortic arch | Q25.21 |
| Total anomalous pulmonary venous return (TAPVR) | Q26.2 |
| Ventricular septal defect (VSD) | Q21.0 |
| Secundum atrial septal defect (ASD) | Q21.1 |
| Other specified defects of septal closure, sinus venosus ASD, inferior sinus venosus ASD, superior sinus venosus ASD | Q21.8 |
| Congenital malformation of cardiac septum, unspecified | Q21.9 |
| Patent ductus arteriosus (PDA) | Q25.0 |
| Aortopulmonary septal defect (AP window) | Q21.4 |
| Partial anomalous pulmonary venous return (PAPVR) | Q26.3 |
| Anomalous pulmonary venous connection, unspecified | Q26.4 |
| Anomalies of the pulmonary valve;  Pulmonary valve anomaly unspecified | Q22.3 |
| Pulmonary valve stenosis (PS) | Q22.1 |
| Pulmonary valve anomaly, other, pulmonary valve regurgitation | Q22.2 |
| Ebstein anomaly of the tricuspid valve | Q22.5 |
| Congenital malformations of tricuspid valve | Q22.8 |
| Congenital malformation of tricuspid valve, unspecified | Q22.9 |
| Aortic valve stenosis (AS) | Q23.0 |
| Aortic insufficiency or bicuspid or unicuspid aortic valve | Q23.1 |
| Other congenital malformations of aortic and mitral valves | Q23.8 |
| Congenital malformation of aortic and mitral valves, and specified | Q23.9 |
| Mitral stenosis or mitral valve abnormalities | Q23.2 |
| Mitral insufficiency, cleft mitral valve | Q23.3 |
| Subaortic stenosis, subaortic membrane | Q24.4 |
| Infundibular or subvalvular pulmonary stenosis | Q24.3 |
| Coarctation of the aorta | Q25.1 |
| Supravalvular aortic stenosis | Q25.3 |
| Hypoplasia of the aorta | Q25.42 |
| Atresia or stenosis of aorta | Q25.29 |
| Absence and aplasia of aorta | Q25.41 |
| Atresia of pulmonary artery | Q25.5 |
| Pulmonary artery atresia, coarctation, or hypoplasia of the branch pulmonary arteries | Q25.71 |
| Anomalies of the pulmonary artery, other | Q25.79 |
| Cor triatriatum | Q24.2 |
| Coronary artery anomaly, anomalous coronary artery, anomalous left coronary artery off the pulmonary artery (ALCAPA), anomalous right coronary artery off the pulmonary artery (ARCAPA) | Q24.5 |
| Double aortic arch | Q25.45 |
| Right aortic arch component of Vascular ring | Q25.47 |
| Anomalous origin of the subclavian artery component of vascular ring | Q25.48 |

*Abbreviations: PHD = pediatric heart defect; ICD-10-CM = International Classification of Diseases 10th revision, Clinical Modification.*

**Table S2. SDE PHD Element ID Inclusion Criteria.**

| **Element ID** | **Element Name** |
| --- | --- |
| CHOA#063 | Anesthesia Preoperative Evaluation Cardiac Comments |
| CHOA#070 | Anesthesia Preoperative Evaluation Cardiac ECTOPIC ATRIAL TACHYCARDIA |
| CHOA#408 | Anesthesia Preoperative Evaluation Cardiac DISORDERS |
| CHOA#607 | Anesthesia Preoperative Evaluation Cardiac Pulmonary Atresia with Ventricular Septal Defect STATUS |
| CHOA#608 | Anesthesia Preoperative Evaluation Cardiac Pulmonary Atresia with Intact Ventricular Septum STATUS |
| CHOA#609 | Anesthesia Preoperative Evaluation Cardiac SINGLE VENTRICLE STATUS |
| CHOA#610 | Anesthesia Preoperative Evaluation Cardiac DILATED AORTA |
| CHOA#611 | Anesthesia Preoperative Evaluation Cardiac DILATED AORTA STATUS |
| CHOA#612 | Anesthesia Preoperative Evaluation Cardiac DYSPLASTIC AORTIC VALVE |
| CHOA#628 | Anesthesia Preoperative Evaluation Cardiac AORTIC ROOT DILATION |
| CHOA#629 | Anesthesia Preoperative Evaluation Cardiac AORTIC ROOT DILATION STATUS |
| CHOA#630 | Anesthesia Preoperative Evaluation Cardiac SUBAORTIC MEMBRANE |
| CHOA#631 | Anesthesia Preoperative Evaluation Cardiac SUBAORTIC MEMBRANE STATUS |
| CHOA#634 | Anesthesia Preoperative Evaluation Cardiac HYPOPLASTIC AORTIC ARCH STATUS |
| CHOA#647 | Anesthesia Preoperative Evaluation Cardiac HYPOTENSION/Cardiovascular SHOC |
| CHOA#688 | Anesthesia Preoperative Evaluation Cardiac Postural Orthostatic Tachycardia Syndrome |
| CHOA#689 | Anesthesia Preoperative Evaluation Cardiac Dysautonomia |
| CHOA#842 | Anesthesia Preoperative Evaluation Cardiac HYPOTENSION |
| CHOA#901 | Anesthesia Preoperative Evaluation Cardiac SINUS ARRHYTHMIA |
| EPIC#10069 | Diagnoses and Problems - Cardiovascular - HYPERTENSION - HYPERTENSION CONTROL LEVEL |
| EPIC#1282 | FINDINGS - TESTS - CARDIOLOGY TESTS - ECHOCARDIOGRAM - VALVE FINDINGS - MITRAL VALVE FINDINGS - LEAFLET MOTION - SYSTOLIC ANTERIOR MOTION |
| EPIC#13073 | Diagnoses and Problems - Cardiovascular - VALVULAR PROBLEMS/MURMURS - VALVULAR PROBLEMS - BICUSPID AORTIC VALVE |
| EPIC#13286 | Diagnoses and Problems - Cardiovascular - ATRIAL SEPTAL DEFECT - ATRIAL SEPTAL DEFECT STATUS |
| EPIC#13289 | Diagnoses and Problems - Cardiovascular - Congenital HEART DISEASE - ANOMALOUS PULMONARY VENOUS RETURN - ANOMALOUS PULMONARY VENOUS RETURN STATUS |
| EPIC#13292 | Diagnoses and Problems - Cardiovascular - Congenital HEART DISEASE - ATRIOVENTRICULAR CANAL DEFECT - ATRIOVENTRICULAR CANAL DEFECT STATUS |
| EPIC#13295 | Diagnoses and Problems - Cardiovascular - VALVULAR PROBLEMS/MURMURS - VALVULAR PROBLEMS - COARCTATION OF THE AORTA - COARCTATION OF THE AORTA STATUS |
| EPIC#13301 | Diagnoses and Problems - Cardiovascular - Congenital HEART DISEASE - DOUBLE OUTLET RIGHT VENTRICLE - DOUBLE OUTLET RIGHT VENTRICLE STATUS |
| EPIC#13304 | Diagnoses and Problems - Cardiovascular - Congenital HEART DISEASE - EBSTEIN ANOMALY - EBSTEIN ANOMALY STATUS |
| EPIC#13307 | Diagnoses and Problems - Cardiovascular - HEART PROBLEM - HYPOPLASTIC LEFT HEART - HYPOPLASTIC LEFT HEART STATUS |
| EPIC#13310 | Diagnoses and Problems - Cardiovascular - VALVULAR PROBLEMS/MURMURS - VALVULAR PROBLEMS - INTERRUPTED AORTIC ARCH - INTERRUPTED AORTIC ARCH STATUS |
| EPIC#13313 | Diagnoses and Problems - Cardiovascular - Congenital HEART DISEASE - PATENT DUCTUS ARTERIOSUS - PATENT DUCTUS ARTERIOSUS STATUS |
| EPIC#13316 | Diagnoses and Problems - Cardiovascular - Congenital HEART DISEASE - PATENT FORAMEN OVALE - PATENT FORAMEN OVALE STATUS |
| EPIC#13322 | Diagnoses and Problems - Cardiovascular - Congenital HEART DISEASE - TETRALOGY OF FALLOT - TETRALOGY OF FALLOT STATUS |
| EPIC#13325 | Diagnoses and Problems - Cardiovascular - HEART PROBLEM - TRANSPOSITION OF THE GREAT VESSELS - TRANSPOSITION OF THE GREAT VESSELS STATUS |
| EPIC#13328 | Diagnoses and Problems - Cardiovascular - Congenital HEART DISEASE - TRUNCUS ARTERIOSUS - TRUNCUS ARTERIOSUS STATUS |
| EPIC#13331 | Diagnoses and Problems - Cardiovascular - Congenital HEART DISEASE - VENTRICULAR SEPTAL DEFECT - VENTRICULAR SEPTAL DEFECT STATUS |
| EPIC#13334 | Diagnoses and Problems - Cardiovascular - VALVULAR PROBLEMS/MURMURS - VALVULAR PROBLEMS - AORTIC STENOSIS - AORTIC STENOSIS STATUS |
| EPIC#13337 | Diagnoses and Problems - Cardiovascular - VALVULAR PROBLEMS/MURMURS - VALVULAR PROBLEMS - AORTIC INSUFFICIENCY - AORTIC INSUFFICIENCY STATUS |
| EPIC#13340 | Diagnoses and Problems - Cardiovascular - VALVULAR PROBLEMS/MURMURS - VALVULAR PROBLEMS - MITRAL STENOSIS - MITRAL STENOSIS STATUS |
| EPIC#13343 | Diagnoses and Problems - Cardiovascular - VALVULAR PROBLEMS/MURMURS - VALVULAR PROBLEMS - MITRAL REGURGITATION - MITRAL REGURGITATION STATUS |
| EPIC#13346 | Diagnoses and Problems - Cardiovascular - VALVULAR PROBLEMS/MURMURS - VALVULAR PROBLEMS - MITRAL VALVE PROLAPSE - MITRAL VALVE PROLAPSE STATUS |
| EPIC#13349 | Diagnoses and Problems - Cardiovascular - VALVULAR PROBLEMS/MURMURS - VALVULAR PROBLEMS - PULMONIC STENOSIS - PULMONIC STENOSIS STATUS |
| EPIC#13355 | Diagnoses and Problems - Cardiovascular - VALVULAR PROBLEMS/MURMURS - VALVULAR PROBLEMS - TRICUSPID REGURGITATION - TRICUSPID REGURGITATION STATUS |
| EPIC#14571 | Diagnoses and Problems - Cardiovascular - HYPERTENSION - HYPERTENSION TYPE - PULMONARY HYPERTENSION |
| EPIC#1532 | Diagnoses and Problems - Cardiovascular - ATRIAL FIBRILLATION |
| EPIC#20282 | Diagnoses and Problems - Cardiovascular - VALVULAR PROBLEMS/MURMURS - VALVULAR PROBLEMS - TRICUSPID REGURGITATION - TRICUSPID REGURGITATION SEVERITY |
| EPIC#20283 | Diagnoses and Problems - Cardiovascular - VALVULAR PROBLEMS/MURMURS - VALVULAR PROBLEMS - TRICUSPID STENOSIS - TRICUSPID STENOSIS SEVERITY |
| EPIC#20286 | Diagnoses and Problems - Cardiovascular - VALVULAR PROBLEMS/MURMURS - VALVULAR PROBLEMS - PULMONIC STENOSIS - PULMONIC STENOSIS SEVERITY |
| EPIC#27028 | PROCEDURAL - SURGICAL HISTORY - SHUNT - INTRACardiac SHUNT |
| EPIC#276 | Diagnoses and Problems - Cardiovascular - MYOCARDITIS |
| EPIC#38126 | Diagnoses and Problems - Cardiovascular - Congenital HEART DISEASE - DOUBLE INLET LEFT VENTRICLE |
| EPIC#38127 | Diagnoses and Problems - Cardiovascular - Congenital HEART DISEASE - ATRIOVENTRICULAR CANAL DEFECT - ATRIOVENTRICULAR CANAL DEFECT TYPE |
| EPIC#38130 | Diagnoses and Problems - Cardiovascular - VALVULAR PROBLEMS/MURMURS - VALVULAR PROBLEMS - PULMONIC VALVE PROBLEMS - PULMONARY ATRESIA (IVS) |
| EPIC#38131 | Diagnoses and Problems - Cardiovascular - VALVULAR PROBLEMS/MURMURS - VALVULAR PROBLEMS - PULMONIC VALVE PROBLEMS - PULMONARY ATRESIA (VSD) |
| EPIC#38132 | PROCEDURAL - SURGICAL HISTORY - TRANSPLANT - HEART TRANSPLANT |
| EPIC#4218 | Diagnoses and Problems - Genetic Disorders - DIGEORGE SEQUENCE |
| EPIC#4233 | Diagnoses and Problems - Genetic Disorders - TrisomyMY 21 |
| EPIC#4239 | Diagnoses and Problems - Genetic Disorders - WILLIAMS SYNDROME |
| EPIC#4276 | Diagnoses and Problems - Cardiovascular - VASCULAR RING |
| EPIC#4360 | Diagnoses and Problems - Cardiovascular - POLYARTERITIS NODOSA |
| EPIC#4372 | Diagnoses and Problems - Cardiovascular - CARDIOMYOPATHY |
| EPIC#4375 | Diagnoses and Problems - Cardiovascular - DECREASED MYOCARDIAL FUNCTION |
| EPIC#4376 | Diagnoses and Problems - Cardiovascular - EISENMENGER SYNDROME |
| EPIC#4378 | Diagnoses and Problems - Cardiovascular - ATRIAL SEPTAL DEFECT |
| EPIC#4380 | Diagnoses and Problems - Cardiovascular - Congenital HEART DISEASE - ATRIOVENTRICULAR CANAL DEFECT |
| EPIC#4381 | Diagnoses and Problems - Cardiovascular - VALVULAR PROBLEMS/MURMURS - VALVULAR PROBLEMS - COARCTATION OF THE AORTA |
| EPIC#4382 | Diagnoses and Problems - Cardiovascular - HEART DISEASE - COR PULMONALE |
| EPIC#4383 | Diagnoses and Problems - Cardiovascular - Congenital HEART DISEASE - COR TRIATRIATUM |
| EPIC#4384 | Diagnoses and Problems - Cardiovascular - Congenital HEART DISEASE - DOUBLE OUTLET RIGHT VENTRICLE |
| EPIC#4385 | Diagnoses and Problems - Cardiovascular - Congenital HEART DISEASE - EBSTEIN ANOMALY |
| EPIC#4386 | Diagnoses and Problems - Cardiovascular - HEART PROBLEM - HYPOPLASTIC LEFT HEART |
| EPIC#4387 | Diagnoses and Problems - Cardiovascular - VALVULAR PROBLEMS/MURMURS - VALVULAR PROBLEMS - INTERRUPTED AORTIC ARCH |
| EPIC#4388 | Diagnoses and Problems - Cardiovascular - Congenital HEART DISEASE - PATENT DUCTUS ARTERIOSUS |
| EPIC#4389 | Diagnoses and Problems - Cardiovascular - Congenital HEART DISEASE - PATENT FORAMEN OVALE |
| EPIC#4390 | Diagnoses and Problems - Cardiovascular - Congenital HEART DISEASE - SHONE COMPLEX |
| EPIC#4391 | Diagnoses and Problems - Cardiovascular - Congenital HEART DISEASE - TETRALOGY OF FALLOT |
| EPIC#4392 | Diagnoses and Problems - Cardiovascular - HEART PROBLEM - TRANSPOSITION OF THE GREAT VESSELS |
| EPIC#4393 | Diagnoses and Problems - Cardiovascular - Congenital HEART DISEASE - TRUNCUS ARTERIOSUS |
| EPIC#4394 | Diagnoses and Problems - Cardiovascular - Congenital HEART DISEASE - VENTRICULAR SEPTAL DEFECT |
| EPIC#4395 | Diagnoses and Problems - Cardiovascular - VALVULAR PROBLEMS/MURMURS - VALVULAR PROBLEMS - AORTIC STENOSIS |
| EPIC#4396 | Diagnoses and Problems - Cardiovascular - VALVULAR PROBLEMS/MURMURS - VALVULAR PROBLEMS - AORTIC INSUFFICIENCY |
| EPIC#4397 | Diagnoses and Problems - Cardiovascular - VALVULAR PROBLEMS/MURMURS - VALVULAR PROBLEMS - MITRAL STENOSIS |
| EPIC#4398 | Diagnoses and Problems - Cardiovascular - VALVULAR PROBLEMS/MURMURS - VALVULAR PROBLEMS - MITRAL REGURGITATION |
| EPIC#4399 | Diagnoses and Problems - Cardiovascular - VALVULAR PROBLEMS/MURMURS - VALVULAR PROBLEMS - PULMONIC STENOSIS |
| EPIC#4418 | Diagnoses and Problems - Cardiovascular - VALVULAR PROBLEMS/MURMURS - VALVULAR PROBLEMS - TRICUSPID STENOSIS |
| EPIC#4419 | Diagnoses and Problems - Cardiovascular - VALVULAR PROBLEMS/MURMURS - VALVULAR PROBLEMS - TRICUSPID REGURGITATION |
| EPIC#4424 | Diagnoses and Problems - Cardiovascular - ENDOCARDITIS |
| EPIC#4425 | Diagnoses and Problems - Cardiovascular - HEART PROBLEM - PERICARDIAL EFFUSION |
| EPIC#4426 | Diagnoses and Problems - Cardiovascular - HEART PROBLEM - PERICARDIAL NEOPLASM |
| EPIC#4427 | Diagnoses and Problems - Cardiovascular - HEART PROBLEM - PERICARDIAL TAMPONADE |
| EPIC#4428 | Diagnoses and Problems - Cardiovascular - HEART PROBLEM - PERICARDITIS |
| EPIC#4429 | Diagnoses and Problems - Cardiovascular - HEART PROBLEM - SUPERIOR VENA CAVA SYNDROME |
| EPIC#4432 | Diagnoses and Problems - Cardiovascular - HEART PROBLEM - HEART BLOCK |
| EPIC#4433 | Diagnoses and Problems - Cardiovascular - HEART PROBLEM - ATRIAL FLUTTER |
| EPIC#4434 | Diagnoses and Problems - Cardiovascular - HEART PROBLEM - JUNCTIONAL ECTOPIC TACHYCARDIA |
| EPIC#4435 | Diagnoses and Problems - Cardiovascular - HEART PROBLEM - LONG QT SYNDROME |
| EPIC#4436 | Diagnoses and Problems - Cardiovascular - HEART PROBLEM - PRE-EXCITATION SYNDROME |
| EPIC#4437 | Diagnoses and Problems - Cardiovascular - HEART PROBLEM - PREMATURE VENTRICULAR CONTRACTION |
| EPIC#4438 | Diagnoses and Problems - Cardiovascular - HEART PROBLEM - SICK SINUS SYNDROME |
| EPIC#4439 | Diagnoses and Problems - Cardiovascular - SINUS BRADYCARDIA |
| EPIC#4441 | Diagnoses and Problems - Cardiovascular - SINUS TACHYCARDIA |
| EPIC#4443 | Diagnoses and Problems - Cardiovascular - HEART PROBLEM - VENTRICULAR FIBRILLATION |
| EPIC#4444 | Diagnoses and Problems - Cardiovascular - HEART PROBLEM - VENTRICULAR ECTOPY |
| EPIC#4445 | Diagnoses and Problems - Cardiovascular - HEART PROBLEM - VENTRICULAR TACHYCARDIA |
| EPIC#4474 | Diagnoses and Problems - Cardiovascular - CARDIOMYOPATHY - CARDIOMYOPATHY TYPE |
| EPIC#4475 | Diagnoses and Problems - Cardiovascular - Congenital HEART DISEASE - ANOMALOUS PULMONARY VENOUS RETURN - ANOMALOUS PULMONARY VENOUS RETURN TYPE |
| EPIC#4476 | Diagnoses and Problems - Cardiovascular - VALVULAR PROBLEMS/MURMURS - VALVULAR PROBLEMS - AORTIC STENOSIS - AORTIC STENOSIS SEVERITY |
| EPIC#4477 | Diagnoses and Problems - Cardiovascular - VALVULAR PROBLEMS/MURMURS - VALVULAR PROBLEMS - AORTIC INSUFFICIENCY - AORTIC INSUFFICIENCY SEVERITY |
| EPIC#4478 | Diagnoses and Problems - Cardiovascular - VALVULAR PROBLEMS/MURMURS - VALVULAR PROBLEMS - MITRAL STENOSIS - MITRAL STENOSIS SEVERITY |
| EPIC#4479 | Diagnoses and Problems - Cardiovascular - VALVULAR PROBLEMS/MURMURS - VALVULAR PROBLEMS - MITRAL REGURGITATION - MITRAL REGURGITATION SEVERITY |
| EPIC#4480 | Diagnoses and Problems - Cardiovascular - HEART PROBLEM - HEART BLOCK - HEART BLOCK TYPE |
| EPIC#4481 | EPIC RETIRED SMARTDATA ELEMENTS - PRE-EXCITATION SYNDROME TYPE |
| EPIC#4652 | Diagnoses and Problems - Cardiovascular - Congenital HEART DISEASE - ANOMALOUS PULMONARY VENOUS RETURN |
| EPIC#47322 | EPIC USE ONLY - CARDIOLOGY - ECHO FINDINGS - AORTA - AORTIC ARCH - HYPOPLASTIC AORTIC ARCH |
| EPIC#49547 | Diagnoses and Problems - Cardiovascular - Congenital HEART DISEASE - SINGLE VENTRICLE DEFECT |
| EPIC#49548 | Diagnoses and Problems - Cardiovascular - VALVULAR PROBLEMS/MURMURS - VALVULAR PROBLEMS - TRICUSPID VALVE PROBLEMS - TRICUSPID ATRESIA |
| EPIC#49549 | Diagnoses and Problems - Cardiovascular - Congenital HEART DISEASE - MAJOR AORTOPULMONARY COLLATERAL ARTERY |
| EPIC#93917 | FINDINGS - TESTS - CARDIOLOGY TESTS - ECHOCARDIOGRAM - IVC FINDINGS - INFERIOR VENA CAVA - Congenital ANOMALIES - HETEROTAXY |
| EPIC#96591 | FINDINGS - TESTS - CARDIOLOGY TESTS - CATHETERIZATION - MYOCARDIAL - Congenital DEFECTS - APEX POSITION - DEXTROCARDIA |
| EPIC#CVES0006 | FINDINGS - TESTS - CARDIOLOGY TESTS - ELECTROCARDIOGRAM - ECTOPY - ATRIAL PREMATURE CONTRACTIONS |
| EPIC#HPI0094 | Diagnoses and Problems - Cardiovascular - HYPERTENSION |
| EPIC#HPI0116 | Diagnoses and Problems - Cardiovascular - HEART FAILURE |
| EPIC#HPI0330 | Diagnoses and Problems - Cardiovascular - VALVULAR PROBLEMS/MURMURS - VALVULAR PROBLEMS - MITRAL VALVE PROLAPSE |
| EPIC#HPI0332 | Diagnoses and Problems - Cardiovascular - ARRHYTHMIA - SVT |
| EPIC#HPI0333 | PROCEDURAL - CARDIOLOGY - PACEMAKER |
| EPIC#RSCV0008 | SYMPTOMS - CONSTITUTIONAL - SYNCOPE |
| EPIC#RSSK0014 | Diagnoses and Problems - Cardiovascular - CYANOSIS |
| LCAN#0010 | Anesthesia Preoperative Evaluation Cardiac HEART FAILURE TYPE |
| LCAN#0024 | Anesthesia Preoperative Evaluation Cardiac ANTHRACYCLINE EXPOSURE |
| LCAN#0027 | Anesthesia Preoperative Evaluation Cardiac AORTIC STENOSIS LOCATION |
| LCAN#0028 | Anesthesia Preoperative Evaluation Cardiac HYPOPLASTIC RIGHT HEART |
| LCAN#0033 | Anesthesia Preoperative Evaluation Cardiac UNROOFED CORONARY SINUS |
| LCAN#0034 | Anesthesia Preoperative Evaluation Cardiac LEFT SVC |
| LCAN#0035 | Anesthesia Preoperative Evaluation Cardiac RIGHT AORTIC ARCH |
| LCAN#0037 | Anesthesia Preoperative Evaluation Cardiac ANOMALOUS ORIGIN OF THE CORONARY ARTERIES |
| LCAN#0039 | Anesthesia Preoperative Evaluation Cardiac MITRAL VALVE CLEFT |
| LCAN#0040 | Anesthesia Preoperative Evaluation Cardiac PULMONIC REGURGITATION |
| LCAN#0042 | Anesthesia Preoperative Evaluation Cardiac HYPOPLASTIC RIGHT HEART TYPE |
| LCAN#0045 | Anesthesia Preoperative Evaluation Cardiac TRICUSPID ATRESIA TYPE |
| LCAN#0048 | Anesthesia Preoperative Evaluation Cardiac MITRAL VALVE ARCADE TYPE |
| LCAN#0051 | Anesthesia Preoperative Evaluation Cardiac DOUBLE INLET LEFT VENTRICLE TYPE |
| LCAN#0083 | Anesthesia Preoperative Evaluation Cardiac PULMONIC REGURG TYPE |
| LCAN#0084 | Anesthesia Preoperative Evaluation Cardiac PULMONIC REGURG STATUS |
| LCAN#0094 | Anesthesia Preoperative Evaluation Cardiac INNOCENT MURMUR |

*Abbreviations: SDE = smart data element; PHD = pediatric heart defect.*

**Table S3. Model Performance in PHD Detection Evaluated at CHOA.**

| **Model** | **Base Model** | **Size** | **CoT Strategy** | **Average CoT Tokens** | **Training Setup** | **CoT Generation Time (hr)** | **Fine Tuning Time (hr)** | **Inference Time (hr)** |
| --- | --- | --- | --- | --- | --- | --- | --- | --- |
| LLaMA3.2-3B | LLaMA3.2 | 3B | N/A | 0 | LoRA (r=16, 4-bit) | N/A | 0.05 | 2.5 |
| LLaMA3.2-3B-391-otk | LLaMA3.2 | 3B | Overthinking CoT | 392 | LoRA (r=16, 4-bit) | 0.16 | 0.05 | 2.8 |
| LLaMA3.2-3B-1382-otk | LLaMA3.2 | 3B | Overthinking CoT | 1.4k | LoRA (r=16, 4-bit) | 0.50 | 0.05 | 3.2 |
| LLaMA3.2-3B-2635-otk | LLaMA3.2 | 3B | Overthinking CoT | 2.6k | LoRA (r=16, 4-bit) | 1.00 | 0.05 | 3.8 |
| LLaMA3.2-3B-4678-otk | LLaMA3.2 | 3B | Overthinking CoT | 4.7k | LoRA (r=16, 4-bit) | 2.00 | 0.05 | 4.5 |
| LLaMA3.2-3B-10174-otk | LLaMA3.2 | 3B | Overthinking CoT | 10k | LoRA (r=16, 4-bit) | 4.00 | 0.05 | 5.5 |
| Qwen2.5-3B | Qwen2.5 | 3B | N/A | 0 | LoRA (r=16, 4-bit) | N/A | 0.05 | 2.8 |
| Qwen2.5-3B-391.96-otk | Qwen2.5 | 3B | Overthinking CoT | 392 | LoRA (r=16, 4-bit) | 0.25 | 0.05 | 3.0 |
| Qwen2.5-3B-1382.55-otk | Qwen2.5 | 3B | Overthinking CoT | 1.4k | LoRA (r=16, 4-bit) | 0.67 | 0.05 | 3.5 |
| Qwen2.5-3B-2635.99-otk | Qwen2.5 | 3B | Overthinking CoT | 2.6k | LoRA (r=16, 4-bit) | 1.25 | 0.05 | 4.0 |
| Qwen2.5-3B-4678.44-otk | Qwen2.5 | 3B | Overthinking CoT | 4.7k | LoRA (r=16, 4-bit) | 2.33 | 0.05 | 4.8 |
| Qwen2.5-3B-10174.95-otk | Qwen2.5 | 3B | Overthinking CoT | 10k | LoRA (r=16, 4-bit) | 4.67 | 0.05 | 6.0 |
| Qwen2.5-7B | Qwen2.5 | 7B | N/A | 0 | LoRA (r=16, 4-bit) | N/A | 0.05 | 3.5 |
| Qwen2.5-7B-391.96-otk | Qwen2.5 | 7B | Overthinking CoT | 392 | LoRA (r=16, 4-bit) | 0.33 | 0.05 | 4.0 |
| Qwen2.5-7B-1382.55-otk | Qwen2.5 | 7B | Overthinking CoT | 1.4k | LoRA (r=16, 4-bit) | 1.00 | 0.05 | 4.8 |
| Qwen2.5-7B-2635.99-otk | Qwen2.5 | 7B | Overthinking CoT | 2.6k | LoRA (r=16, 4-bit) | 2.00 | 0.05 | 5.5 |
| Qwen2.5-7B-4678.44-otk | Qwen2.5 | 7B | Overthinking CoT | 4.7k | LoRA (r=16, 4-bit) | 3.33 | 0.05 | 6.0 |
| Qwen2.5-7B-10174.95-otk | Qwen2.5 | 7B | Overthinking CoT | 10k | LoRA (r=16, 4-bit) | 5.00 | 0.05 | 6.2 |

**Notes.** This table summarizes the model configurations and computational requirements for pediatric heart disease (PHD) detection using large language models (LLMs) with and without chain-of-thought (CoT) reasoning. All models were fine-tuned using parameter-efficient Low-Rank Adaptation (LoRA; r=16, α=16) with 4-bit quantization on a subset of 71 adjudicated echocardiogram reports. CoT-enhanced models incorporate overthinking CoT prompts with varying average token lengths, generated using the DeepSeek-R1-Distill-Qwen-7B model. CoT generation time increases approximately linearly with token length and represents the primary computational cost, whereas fine-tuning time remains minimal due to parameter-efficient training. Inference time reflects evaluation across the held-out evaluation set (90%, n=641) samples with 10 runs per sample. All experiments were conducted under a consistent hardware environment.

*Abbreviations: CoT = Chain of Thought; LLM = Large Language Model; LoRA = Low-Rank Adaptation; PHD = Pediatric Heart Disease; otk = Overthinking CoT.*

**Table S4. Model Performance in PHD Detection Evaluated at CHOA.**

| ***Characteristic Mean (95% CI)*** | ***CHOA*** | | | | | | | | | | | | | | | | | |
| --- | --- | --- | --- | --- | --- | --- | --- | --- | --- | --- | --- | --- | --- | --- | --- | --- | --- | --- |
| ***Model*** | ***Llama3.2-3B*** | ***Llama3.2-3B-391.96-otk*** | ***Llama3.2-3B-1382.55-otk*** | ***Llama3.2-3B-2635.99-otk*** | ***Llama3.2-3B-4678.44-otk*** | ***Llama3.2-3B-10174.95-otk*** | ***Qwen2.5-3B*** | ***Qwen2.5-3B-391.96-otk*** | ***Qwen2.5-3B-1382.55-otk*** | ***Qwen2.5-3B-2635.99-otk*** | ***Qwen2.5-3B-4678.44-otk*** | ***Qwen2.5-3B-10174.95-otk*** | ***Qwen2.5-7B*** | ***Qwen2.5-7B-391.96-otk*** | ***Qwen2.5-7B-1382.55-otk*** | ***Qwen2.5-7B-2635.99-otk*** | ***Qwen2.5-7B-4678.44-otk*** | ***Qwen2.5-7B-10174.95-otk*** |
| ***Accuracy*** | ***0.879***  ***(0.855, 0.902)*** | ***0.912***  ***(0.888, 0.930)*** | ***0.811***  ***(0.789, 0.845)*** | ***0.696***  ***(0.665, 0.732)*** | ***0.617***  ***(0.580, 0.652)*** | ***0.462***  ***(0.391, 0.464)*** | ***0.856***  ***(0.828, 0.879)*** | ***0.856***  ***(0.828, 0.879)*** | ***0.911***  ***(0.888, 0.930)*** | ***0.923***  ***(0.899, 0.939)*** | ***0.883***  ***(0.855, 0.902)*** | ***0.685***  ***(0.650, 0.718)*** | ***0.900***  ***(0.876, 0.920)*** | ***0.915***  ***(0.895, 0.935)*** | ***0.909***  ***(0.890, 0.931)*** | ***0.916***  ***(0.893, 0.934)*** | ***0.924***  ***(0.898, 0.938)*** | ***0.924***  ***(0.902, 0.941)*** |
| ***PPV (Precision)*** | ***0.912***  ***(0.884, 0.934)*** | ***0.924***  ***(0.898, 0.943)*** | ***0.922***  ***(0.893, 0.943)*** | ***0.910***  ***(0.876, 0.936)*** | ***0.875***  ***(0.833, 0.907)*** | ***0.717***  ***(0.655, 0.772)*** | ***0.968***  ***(0.946, 0.981)*** | ***0.968***  ***(0.946, 0.981)*** | ***0.955***  ***(0.933, 0.970)*** | ***0.941***  ***(0.917, 0.958)*** | ***0.938***  ***(0.912, 0.956)*** | ***0.788***  ***(0.749, 0.822)*** | ***0.936***  ***(0.911, 0.954)*** | ***0.961***  ***(0.940, 0.975)*** | ***0.942***  ***(0.918, 0.959)*** | ***0.927***  ***(0.902, 0.947)*** | ***0.933***  ***(0.908, 0.951)*** | ***0.947***  ***(0.923, 0.963)*** |
| ***NPV*** | ***0.801***  ***(0.740, 0.801)*** | ***0.878***  ***(0.824, 0.918)*** | ***0.645***  ***(0.586, 0.700)*** | ***0.489***  ***(0.437, 0.541)*** | ***0.516***  ***(0.369, 0.465)*** | ***0.292***  ***(0.254, 0.334)*** | ***0.693***  ***(0.627, 0.735)*** | ***0.683***  ***(0.627, 0.735)*** | ***0.818***  ***(0.762, 0.863)*** | ***0.871***  ***(0.818, 0.911)*** | ***0.762***  ***(0.703, 0.812)*** | ***0.459***  ***(0.395, 0.525)*** | ***0.817***  ***(0.760, 0.863)*** | ***0.824***  ***(0.769, 0.868)*** | ***0.834***  ***(0.788, 0.886)*** | ***0.884***  ***(0.831, 0.922)*** | ***0.886***  ***(0.833, 0.924)*** | ***0.869***  ***(0.816, 0.908)*** |
| ***Recall (Sensitivity)*** | ***0.919***  ***(0.892, 0.940)*** | ***0.955***  ***(0.933, 0.970)*** | ***0.814***  ***(0.778, 0.846)*** | ***0.640***  ***(0.598, 0.681)*** | ***0.538***  ***(0.494, 0.581)*** | ***0.346***  ***(0.306, 0.388)*** | ***0.826***  ***(0.791, 0.857)*** | ***0.824***  ***(0.789, 0.855)*** | ***0.919***  ***(0.892, 0.940)*** | ***0.949***  ***(0.926, 0.967)*** | ***0.891***  ***(0.861, 0.916)*** | ***0.763***  ***(0.724, 0.798)*** | ***0.923***  ***(0.896, 0.943)*** | ***0.921***  ***(0.894, 0.941)*** | ***0.935***  ***(0.910, 0.953)*** | ***0.957***  ***(0.935, 0.971)*** | ***0.957***  ***(0.935, 0.971)*** | ***0.947***  ***(0.923, 0.963)*** |
| ***Specificity*** | ***0.782***  ***(0.720, 0.833)*** | ***0.806***  ***(0.746, 0.854)*** | ***0.830***  ***(0.773, 0.875)*** | ***0.845***  ***(0.789, 0.888)*** | ***0.811***  ***(0.752, 0.858)*** | ***0.748***  ***(0.684, 0.802)*** | ***0.927***  ***(0.883, 0.955)*** | ***0.932***  ***(0.889, 0.959)*** | ***0.893***  ***(0.844, 0.928)*** | ***0.854***  ***(0.800, 0.896)*** | ***0.854***  ***(0.800, 0.896)*** | ***0.495***  ***(0.428, 0.563)*** | ***0.846***  ***(0.789, 0.888)*** | ***0.908***  ***(0.860, 0.940)*** | ***0.859***  ***(0.805, 0.900)*** | ***0.816***  ***(0.757, 0.863)*** | ***0.830***  ***(0.773, 0.875)*** | ***0.869***  ***(0.816, 0.908)*** |
| ***F1 Score*** | ***0.916***  ***(0.898, 0.934)*** | ***0.939***  ***(0.923, 0.953)*** | ***0.865***  ***(0.841, 0.887)*** | ***0.752***  ***(0.719, 0.784)*** | ***0.666***  ***(0.630, 0.701)*** | ***0.443***  ***(0.397, 0.488)*** | ***0.683***  ***(0.627, 0.735)*** | ***0.890***  ***(0.869, 0.911)*** | ***0.937***  ***(0.920, 0.951)*** | ***0.945***  ***(0.929, 0.959)*** | ***0.914***  ***(0.895, 0.932)*** | ***0.775***  ***(0.747, 0.801)*** | ***0.929***  ***(0.913, 0.944)*** | ***0.940***  ***(0.925, 0.955)*** | ***0.938***  ***(0.922, 0.954)*** | ***0.942***  ***(0.926, 0.956)*** | ***0.944***  ***(0.929, 0.959)*** | ***0.947***  ***(0.933, 0.960)*** |

*Abbreviations: PHD = pediatric heart defect; PPV = Positive Predictive Value; NPV = Negative Predictive Value.*

**Table S5. Model Performance in PHD Detection Evaluated at External Duke Dataset.**

| **Characteristic Mean (95% CI)** | **Duke Validation** | | | | | | | | | | | | | | | | | |
| --- | --- | --- | --- | --- | --- | --- | --- | --- | --- | --- | --- | --- | --- | --- | --- | --- | --- | --- |
| **Model** | ***Llama3.2-3B*** | ***Llama3.2-3B-391.96-otk*** | ***Llama3.2-3B-1382.55-otk*** | ***Llama3.2-3B-2635.99-otk*** | ***Llama3.2-3B-4678.44-otk*** | ***Llama3.2-3B-10174.95-otk*** | ***Qwen2.5-3B*** | ***Qwen2.5-3B-391.96-otk*** | ***Qwen2.5-3B-1382.55-otk*** | ***Qwen2.5-3B-2635.99-otk*** | ***Qwen2.5-3B-4678.44-otk*** | ***Qwen2.5-3B-10174.95-otk*** | ***Qwen2.5-7B*** | ***Qwen2.5-7B-391.96-otk*** | ***Qwen2.5-7B-1382.55-otk*** | ***Qwen2.5-7B-2635.99-otk*** | ***Qwen2.5-7B-4678.44-otk*** | ***Qwen2.5-7B-10174.95-otk*** |
| **Accuracy** | **0.868**  **(0.792, 0.918)** | **0.823**  **(0.752, 0.890)** | **0.810**  **(0.733, 0.875)** | **0.756**  **(0.656, 0.815)** | **0.687**  **(0.609, 0.776)** | **0.462**  **(0.371, 0.552)** | **0.845**  **(0.772, 0.904)** | **0.802**  **(0.713, 0.860)** | **0.814**  **(0.742, 0.882)** | **0.841**  **(0.782, 0.911)** | **0.806**  **(0.713, 0.860)** | **0.589**  **(0.527, 0.704)** | **0.884**  **(0.813, 0.932)** | **0.834**  **(0.752, 0.890)** | **0.865**  **(0.782, 0.911)** | **0.838**  **(0.762, 0.897)** | **0.793**  **(0.703, 0.853)** | **0.827**  **(0.752, 0.890)** |
| **PPV (Precision)** | **0.915**  **(0.816, 0.963)** | **0.909**  **(0.804, 0.961)** | **0.891**  **(0.782, 0.949)** | **0.889**  **(0.765, 0.952)** | **0.857**  **(0.722, 0.933)** | **0.523**  **(0.404, 0.640)** | **0.912**  **(0.811, 0.962)** | **0.918**  **(0.808, 0.968)** | **0.907**  **(0.801, 0.960)** | **0.914**  **(0.814, 0.963)** | **0.918**  **(0.808, 0.968)** | **0.652**  **(0.534, 0.754)** | **0.918**  **(0.822, 0.964)** | **0.881**  **(0.775, 0.941)** | **0.887**  **(0.785, 0.944)** | **0.883**  **(0.778, 0.942)** | **0.833**  **(0.720, 0.907)** | **0.869**  **(0.762, 0.932)** |
| **NPV** | **0.815**  **(0.692, 0.896)** | **0.759**  **(0.635, 0.850)** | **0.741**  **(0.616, 0.837)** | **0.647**  **(0.528, 0.750)** | **0.606**  **(0.489, 0.711)** | **0.375**  **(0.252, 0.516)** | **0.786**  **(0.662, 0.873)** | **0.703**  **(0.582, 0.801)** | **0.746**  **(0.622, 0.839)** | **0.800**  **(0.676, 0.884)** | **0.703**  **(0.582, 0.801)** | **0.568**  **(0.422, 0.703)** | **0.846**  **(0.725, 0.920)** | **0.778**  **(0.651, 0.868)** | **0.824**  **(0.697, 0.904)** | **0.792**  **(0.665, 0.880)** | **0.736**  **(0.604, 0.836)** | **0.788**  **(0.660, 0.878)** |
| **Recall (Sensitivity)** | **0.844**  **(0.736, 0.913)** | **0.781**  **(0.666, 0.865)** | **0.766**  **(0.649, 0.853)** | **0.625**  **(0.503, 0.733)** | **0.562**  **(0.441, 0.677)** | **0.531**  **(0.411, 0.648)** | **0.812**  **(0.700, 0.889)** | **0.703**  **(0.582, 0.801)** | **0.766**  **(0.649, 0.853)** | **0.828**  **(0.718, 0.901)** | **0.703**  **(0.582, 0.801)** | **0.703**  **(0.582, 0.801)** | **0.875**  **(0.772, 0.935)** | **0.812**  **(0.700, 0.889)** | **0.859**  **(0.754, 0.924)** | **0.828**  **(0.718, 0.901)** | **0.781**  **(0.666, 0.865)** | **0.828**  **(0.718, 0.901)** |
| **Specificity** | **0.898**  **(0.782, 0.956)** | **0.898**  **(0.782, 0.956)** | **0.878**  **(0.758, 0.943)** | **0.898**  **(0.782, 0.956)** | **0.878**  **(0.758, 0.943)** | **0.367**  **(0.247, 0.507)** | **0.898**  **(0.782, 0.956)** | **0.918**  **(0.808, 0.968)** | **0.898**  **(0.782, 0.956)** | **0.898**  **(0.782, 0.956)** | **0.918**  **(0.808, 0.968)** | **0.510**  **(0.375, 0.644)** | **0.898**  **(0.782, 0.956)** | **0.857**  **(0.733, 0.929)** | **0.857**  **(0.733, 0.929)** | **0.857**  **(0.733, 0.929)** | **0.796**  **(0.664, 0.885)** | **0.837**  **(0.710, 0.915)** |
| **F1 Score** | **0.878**  **(0.811, 0.932)** | **0.840**  **(0.762, 0.903)** | **0.824**  **(0.743, 0.893)** | **0.734**  **(0.633, 0.819)** | **0.679**  **(0.568, 0.776)** | **0.527**  **(0.416, 0.623)** | **0.860**  **(0.789, 0.918)** | **0.796**  **(0.706, 0.868)** | **0.831**  **(0.745, 0.899)** | **0.869**  **(0.800, 0.925)** | **0.796**  **(0.705, 0.871)** | **0.677**  **(0.574, 0.767)** | **0.896**  **(0.833, 0.947)** | **0.846**  **(0.770, 0.908)** | **0.873**  **(0.807, 0.928)** | **0.855**  **(0.785, 0.914)** | **0.806**  **(0.721, 0.876)** | **0.848**  **(0.772, 0.909)** |

*Abbreviations: PHD = pediatric heart defect; PPV = Positive Predictive Value; NPV = Negative Predictive Value.*

**
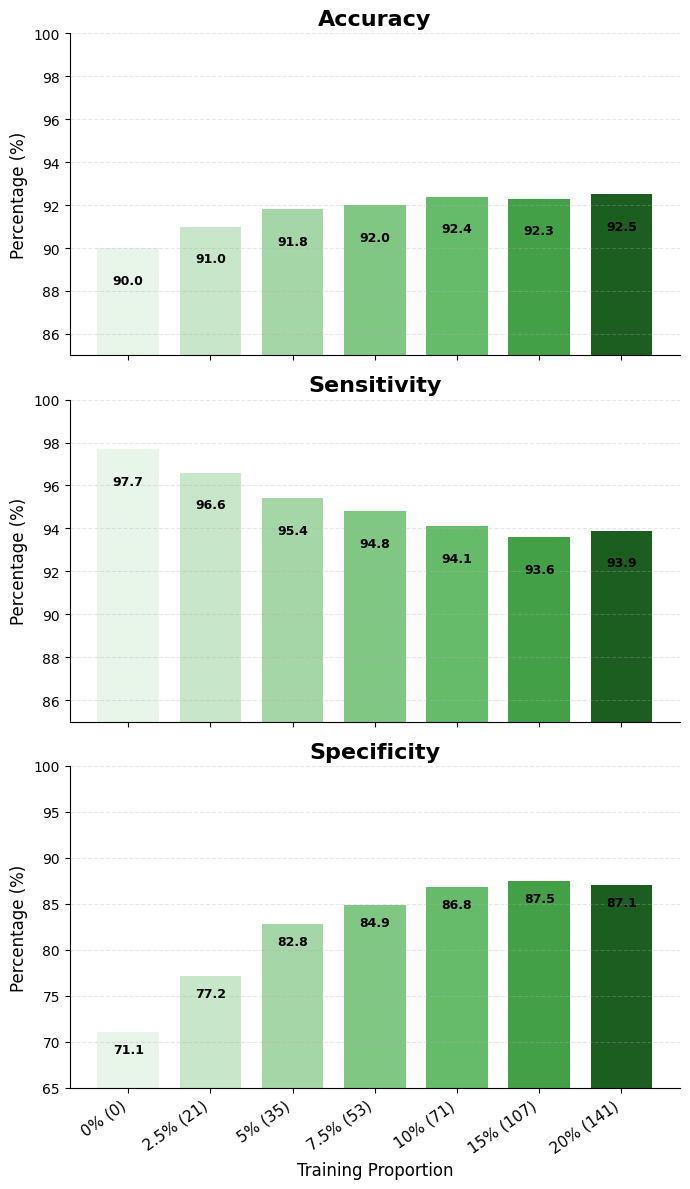
**

**Figure S1. Impact of Training Proportion on Model Performance Using Qwen2.5-7B-10k-Overthinking-CoT Backbone.**

**Notes.** This figure illustrates the effect of varying training data proportions on model performance for clinically significant pediatric heart disease (PHD) detection using the Qwen2.5-7B model with a DeepSeek-R1 generated overthinking chain-of-thought (CoT) prompt (average 10,174.95 tokens). Performance metrics include (a) overall accuracy, (b) sensitivity, and (c) specificity across training proportions ranging from 0% (no fine-tuning) to 20% of the adjudicated CHOA dataset. Accuracy demonstrates improvement with increasing training data, with the largest gains observed between 0% and 5%, followed by a plateau beyond 10%. Sensitivity decreases modestly as training proportion increases, reflecting reduced bias toward positive classification, while specificity improves substantially at lower training proportions and stabilizes thereafter. These results highlight a trade-off between sensitivity and specificity and suggest that approximately 10% fine-tuning provides a practical balance between performance, generalizability, and computational efficiency.

*Abbreviations: CoT = Chain of Thought; PHD = Pediatric Heart Disease.*

**
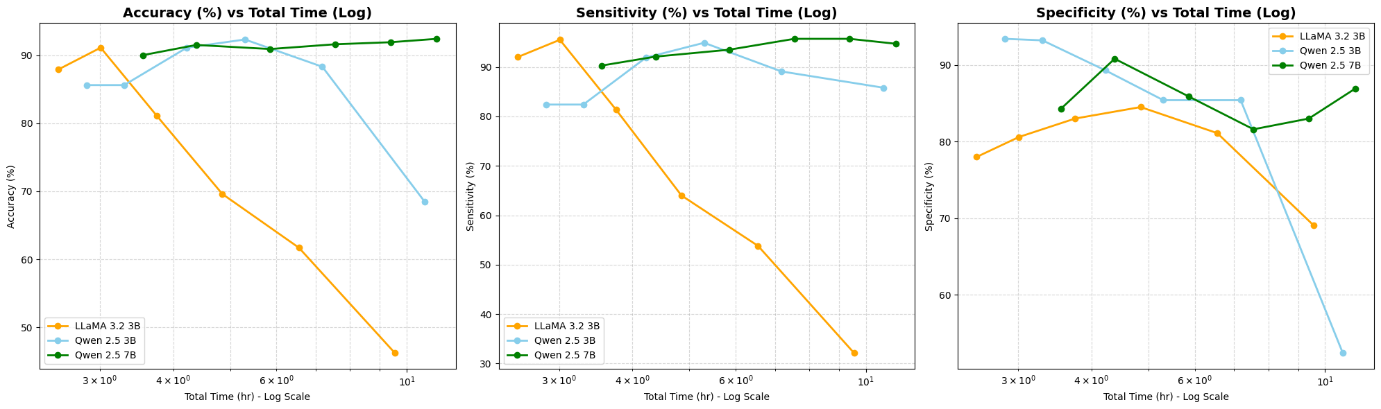
**

**Figure S2. Performance-Computation Trade-off Across Model Architectures and CoT Lengths.**

**Notes.** This figure illustrates the relationship between model performance (accuracy, sensitivity, and specificity) and total computation time (log scale) across different large language models (LLaMA-3.2-3B, Qwen-2.5-3B, and Qwen-2.5-7B) and chain-of-thought (CoT) configurations evaluated on the internal CHOA dataset. Total computation time includes CoT generation, model fine-tuning, and inference. As CoT token length increases, computation time rises substantially across all models. Larger models (Qwen-2.5-7B) maintain relatively stable performance despite increased computational cost, whereas smaller models exhibit performance degradation at longer CoT lengths. These findings demonstrate that increasing reasoning depth introduces significant computational overhead and underscore the importance of balancing CoT length with model capacity and efficiency.

*Abbreviations: CoT = Chain of Thought; LLM = Large Language Model.*

**
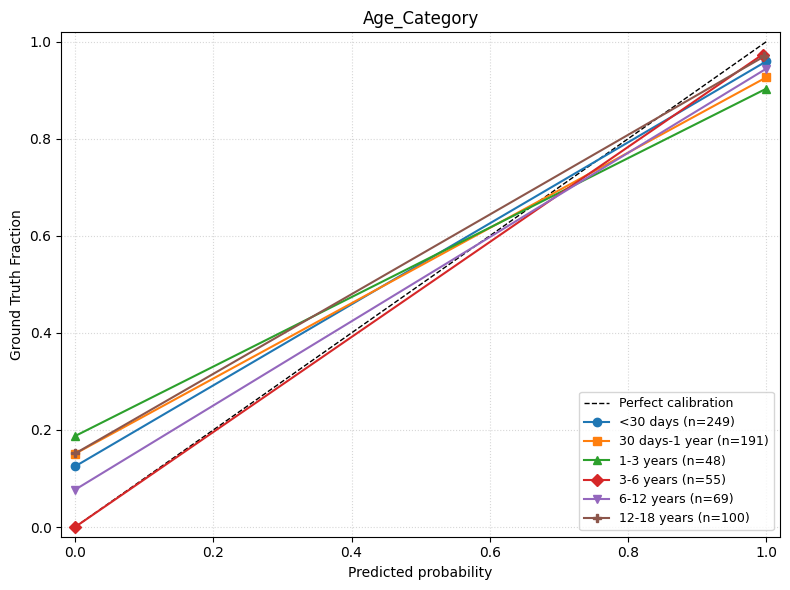
**

**Figure S3. Age-Stratified Calibration of Predicted Probabilities for Clinically Significant PHD.**

**Notes.** This figure presents calibration plots for the best -performing model (Qwen2.5-7B-10k-otk) stratified by patient age group: <30 days (n=249), 30 days–1 year (n=191), 1–3 years (n=48), 3–6 years (n=55), 6–12 years (n=69), and 12–18 years (n=100). The dashed diagonal line represents perfect calibration. Each colored line corresponds to a distinct age category, with markers indicating the mean observed outcome within discrete predicted probability bins. Data points are omitted for probability bins containing fewer than 5 cases to minimize noise and ensure statistical reliability in the visualization.

*Abbreviations: PHD = Pediatric Heart Disease.*


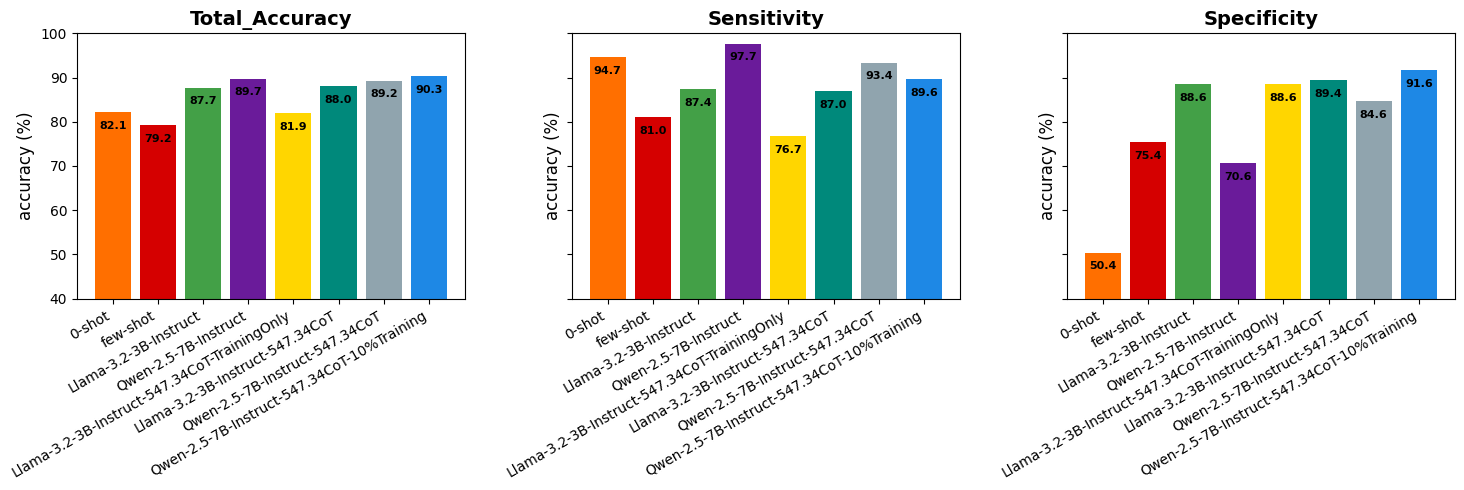


**Figure S4. Comparison of LLM Model Performance in PHD Detection: Accuracy, Sensitivity, and Specificity.**

**Notes.** This figure compares the performance of different Large Language Models (LLMs) for pediatric heart disease (PHD) detection using unstructured echocardiogram reports, highlighting results with and without the application of chain-of-thought (CoT) reasoning. The results are divided into three panels: (a) accuracy, measuring the correctness of predictions for both patients with clinically significant pediatric heart disease and not significant cases; (b) sensitivity for patients with clinically significant pediatric heart disease; and (c) specificity for non-significant pediatric patients. Each of the three subplots shows bar charts for different LLMs, comparing their respective accuracies on each metric. The x-axis lists the various models, and the y-axis indicates percentage accuracy. Each bar’s height reflects how effectively the corresponding model performs in that category.

*Abbreviations: CoT=Chain of Thoughts; LLM=Large Language Model; PHD = Pediatric Heart Disease.*


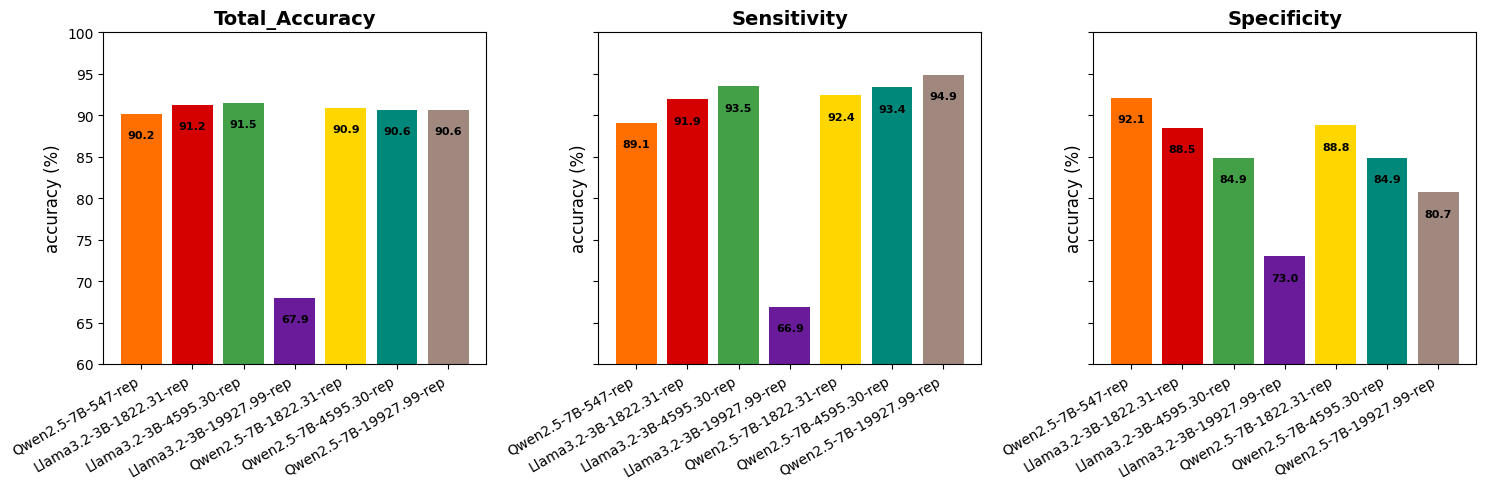


**Figure S5. Comparison of Repetitive CoT LLM Model Performance in PHD Detection: Accuracy, Sensitivity, Specificity.**

**Notes.** This figure compares the performance of different Large Language Models (LLMs) for pediatric heart disease (PHD) detection using unstructured echocardiogram reports with repetitive CoT. The results are divided into three panels: (a) accuracy, measuring the correctness of predictions for both patients with clinically significant pediatric heart disease and not significant cases; (b) sensitivity for patients with clinically significant pediatric heart disease; and (c) specificity for non-significant pediatric patients. Each of the three subplots shows bar charts for different LLMs, comparing their respective accuracies on each metric. The x-axis lists the various models, and the y-axis indicates percentage accuracy. Each bar’s height reflects how effectively the corresponding model performs in that category.

*Abbreviations: CoT=Chain of Thoughts; LLM=Large Language Model; PHD = Pediatric Heart Disease; rep=Repetitive CoT.*


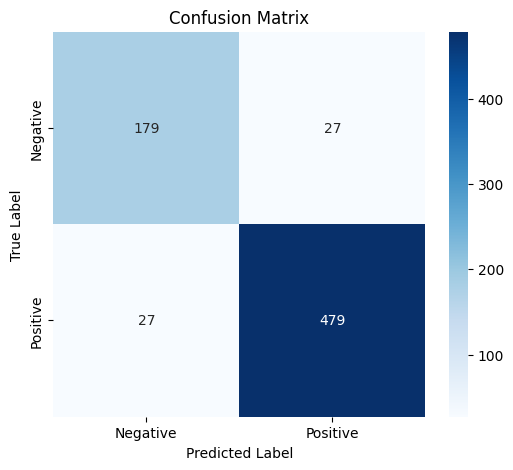


**Figure S6. Confusion matrix for the Qwen-2.5-7B-Instruct-10174.95-Token-otk-CoT-10%-Training model.**

**Notes.** Confusion matrix for the Qwen-2.5-7B-Instruct-10174.95-Token-otk-CoT-10%-Training model in detecting clinically significant PHD. This model was fine-tuned using the Qwen 2.5 7B with an average overthinking chain-of-thought (otk-CoT) token length of 10,174.95, applied to 10% of the training samples (71 samples). The matrix compares the model’s predicted label (positive vs. negative) with the true label for each echocardiogram encounter, highlighting true positives (lower-right), false positives (upper-right), false negatives (lower-left), and true negatives (upper-left).

*Abbreviations: PHD = Pediatric Heart Disease.*


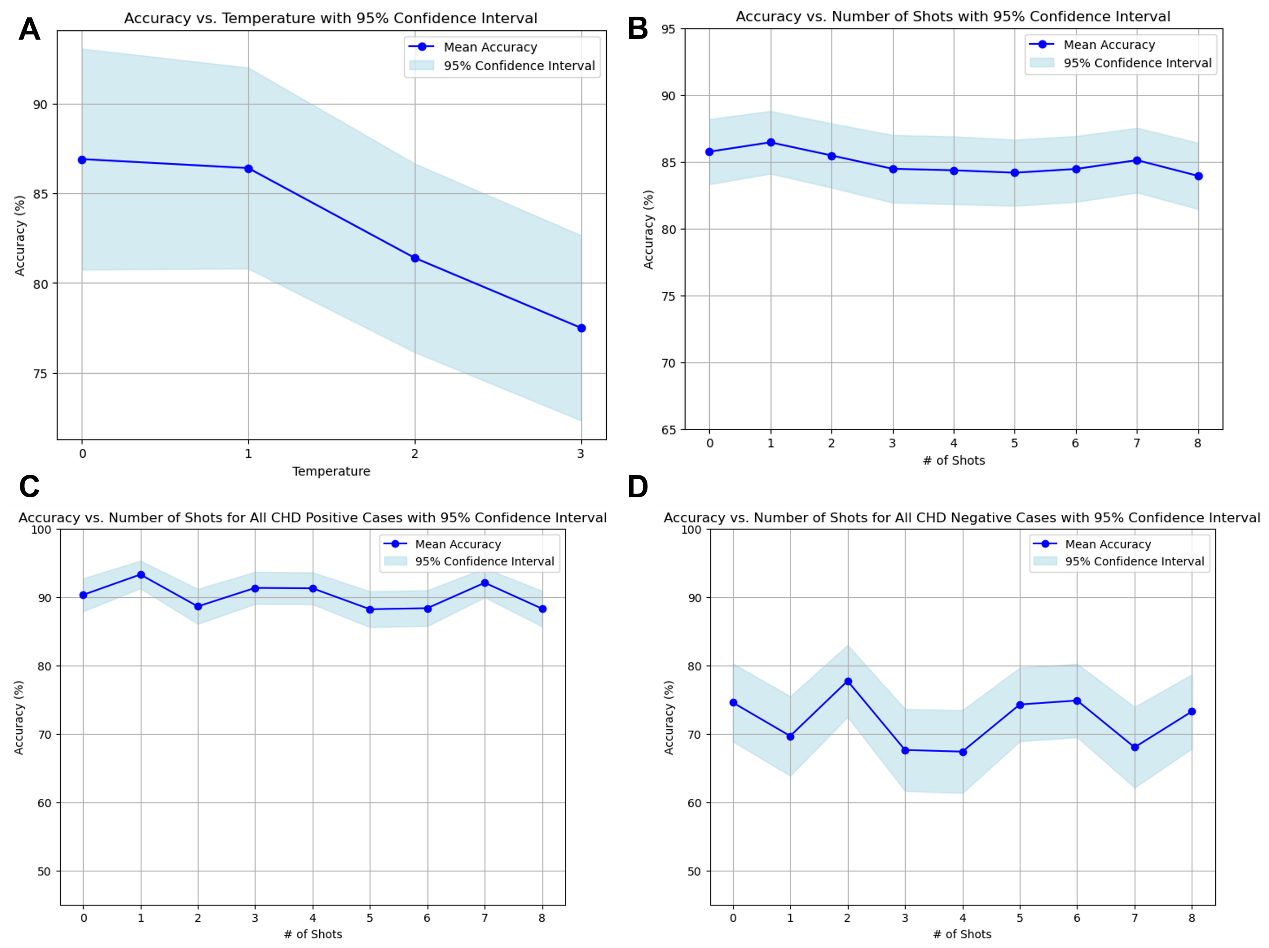


**Figure S7. LLaMA 3.2 3B Model Performance with Different Temperature, # of Shots Learning.**

**Notes.** LLaMA 3.2 3B model performance under varying temperature and shot-based learning conditions with 95% confidence intervals. (a) accuracy vs. temperature, where temperature settings are mapped as follows: 0 = 0.1, 1 = 0.3, 2 = 0.5, 3 = 0.7.; (b) accuracy vs. number of shots (0–8) for both patients with clinically significant pediatric heart disease and not significant cases; (c) accuracy vs. number of shots (0–8) specifically for clinically significant pediatric heart disease; (d) accuracy vs. number of shots (0–8) specifically for non-significant pediatric patients. Each subplot highlights mean accuracy (blue line) and the 95% confidence interval (shaded region).

**
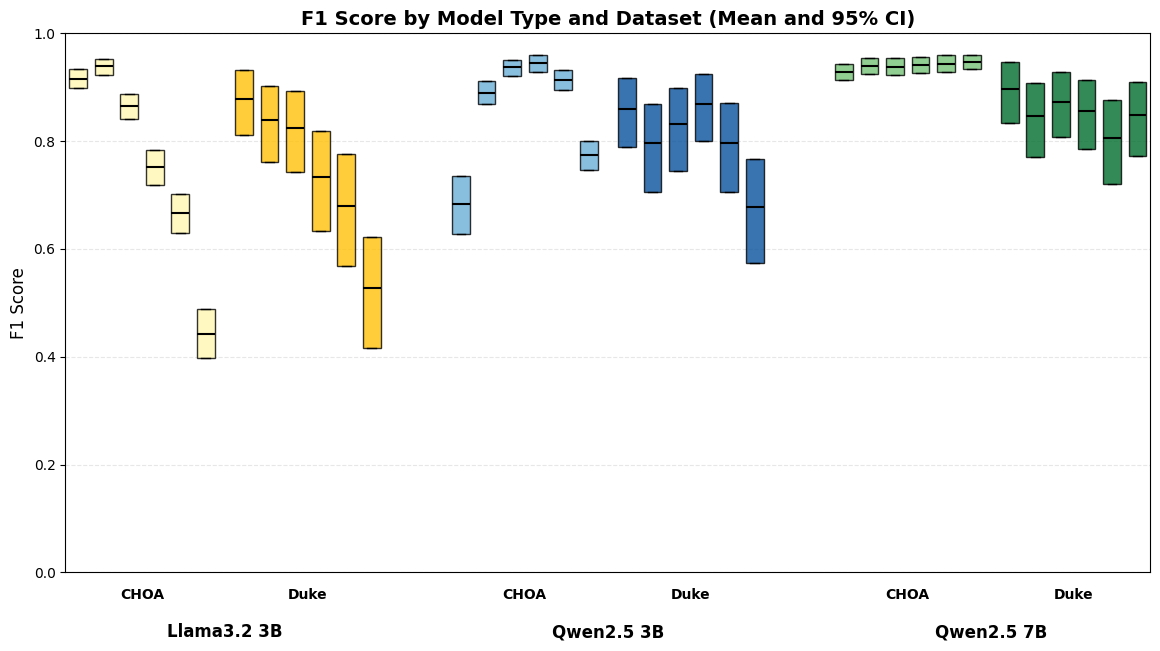
**

**Figure S8. Distribution of F1 Scores Across Model Architectures and Institutional Cohorts.**

**Notes.** This figure displays the distribution of F1 scores (mean and 95% confidence intervals) for LLaMA-3.2-3B, Qwen-2.5-3B, and Qwen-2.5-7B variants evaluated on the internal CHOA dataset and the external Duke validation cohort. Each plot captures the performance variability across ten independent inference runs for every model configuration. The figure highlights differences in model performance between training and external validation sites, demonstrating how site-specific fine-tuning influences the model’s ability to balance precision and recall across differing clinical reporting styles.

*Abbreviations: PHD = Pediatric Heart Disease.*
