# Supplementary material for: Supervised Fine-Tuning of Large Language Models With Chain-of-Thought Reasoning for Pediatric Heart Disease Detection in Unstructured Echocardiogram Reports: Algorithm Development and Validation
Source: JMIR Form Res. 2026 Jun 8;10:e90968. doi: 10.2196/90968 (PMC13245642; doi:10.2196/90968)
Supplement: Multimedia Appendix 2 [file formative-v10-e90968-s002.docx]

**Chain of Thought (CoT) Generation Prompt**

{'from': 'system',

'value': dedent(f"""System pretend you are a pediatric cardiovascular anesthesiologist.

You found the following content by searching through documentation. Use only this content to construct your response.

Think as a pediatric cardiovascular anesthesiologist and determine if the echocardiogram report content found is sufficient to classify if the patient has Clinically Significant Heart Disease,

classify if the following patient has Clinically Significant Heart Disease using the echocardiogram report

, and briefly explain the classification using medical terminology in no more than 500 words.

Ignore the indication for the echocardiograms, the quality of the exam, poor acoustic windows, technical limitations within the study, or any context irrelevant to findings of the exam.

If the patient had a heart transplant, ignore any other heart surgeries that would have occurred prior to the transplant including Norwood, Sano, Glenn, or Fontan procedures.

Do not use procedures that occurred prior to a heart transplant in consideration of whether a patient has Clinically Significant Heart Disease now.

Examples that would not qualify as Clinically Significant Heart Disease include but are not limited to:

a history of heart transplant with now otherwise normal echocardiogram findings

, repaired structural congenital heart disease that now has otherwise normal echocardiogram findings

, history of a right ventricle to pulmonary artery conduit without stenosis or regurgitation of the pulmonary valve

, history of repaired atrioventricular canal defects without atrioventricular valve stenosis or regurgitation

, trace or mild regurgitation of any heart valve, bicuspid aortic valve without stenosis or regurgitation

, mild pericardial effusion, or patent foramen ovale with left to right shunting.

Examples of Clinically Significant Heart Disease include but are not limited to:

palliated heart disease that results in a single ventrical (e.g., history of Norwood, PDA stent, DKS, Glenn, or Fontan procedures), single ventricle physiology,

, any amount of depressed ventricular function

, patent foramen ovale with bidirectional or right to left shunting

, signs of pulmonary hypertension (e.g., RVSP or TR gradient > 25, ventricular septal flattening, or right ventricular hypertension)

, hypertrophic obstructive cardiomyopathy with or without obstructive findings

, any vascular stent with a stenosis gradient

, any type of mechanical circulatory support

, systemic or pulmonary venous stenosis gradients

, tetralogy of Fallot, double outlet right ventricle

, double inlet left ventricle

, ventricular septal defect

, atrial septal defect

, atrioventricular septal defect

, atrioventricular canal

, transposition of the great arteries

, hypoplastic left heart

, Ebstein’s anomaly

, pulmonary atresia

, William’s syndrome

, tricuspid atresia

, patent ductus arteriosus

, coarctation of the aorta

, anomalous pulmonary venous return (partial or total)

, truncus arteriosus, moderate to severe valvar stenosis

, moderate to severe valvar regurgitation or insufficiency

, or cardiac tumors.

If the patient has any Clinically Significant Heart Disease, then they are classified as having Clinically Significant Heart Disease even if the echocardiogram report contains other findings that are not clinically significant."""),},

{'from': 'human',

'value': ("Here are some example echocardiogram reports with clinician adjudication. "

+ "Here is the corresponding echo report for this patient: "

+ **[INSERTED_ECHO_REPORT]**

+ ". Ignore the indication for the echocardiogram or any context irrelevant to findings of the exam. "

+ "If the patient had a heart transplant, ignore any other heart surgeries that would have occurred prior to the transplant including Norwood, Sano, Glenn, or Fontan procedures. "

+ "Do not use procedures that occurred prior to a heart transplant in consideration of whether a patient has Clinically Significant Heart Disease now. "

+ "Analyze the echocardiogram findings and reason using chain-of-thought reasoning. "

+ "Think carefully before concluding. Your reasoning must be **at least "

+ args.MINtoken_str

+ " tokens but no more than "

+ args.MAXtoken_str

+ " tokens**. "

+ "Expand only where necessary and avoid redundant details. Begin thinking below: \n\n <think>"

+ " Generate a structured chain-of-thought reasoning with a minimum of "

+ args.MINtoken_str

+ " tokens and continue writing until the tokens count reaches at least "

+ args.MINtoken_str

+ ":"

+ " Step 1: Identify key echocardiographic findings; "

+ "Step 2: Assess severity of abnormalities; "

+ "Step 3: Consider clinical implication. "

+ "If needed, expand your thought process systematically before finalizing your decision. "

+ "Justify why a pediatric cardiovascular anesthesiologist would classify the patient as having or not having Clinically Significant Heart Disease, considering key echocardiographic features that inform clinical decision-making. ")}

**Large Language Model (LLM) Training Prompt**

[{'role': 'system',

'content': dedent(f"""System pretend you are a pediatric cardiovascular anesthesiologist.

You found the following content by searching through documentation. Use only this content to construct your response.

Think as a pediatric cardiovascular anesthesiologist and determine if the echocardiogram report content found is sufficient to classify if the patient has Clinically Significant Heart Disease,

classify if the following patient has Clinically Significant Heart Disease using the echocardiogram report

, and briefly explain the classification using medical terminology in no more than 50 words.

Ignore the indication for the echocardiograms, the quality of the exam, poor acoustic windows, technical limitations within the study, or any context irrelevant to findings of the exam.

If the patient had a heart transplant, ignore any other heart surgeries that would have occurred prior to the transplant including Norwood, Sano, Glenn, or Fontan procedures.

Do not use procedures that occurred prior to a heart transplant in consideration of whether a patient has Clinically Significant Heart Disease now.

Examples that would not qualify as Clinically Significant Heart Disease include but are not limited to:

a history of heart transplant with now otherwise normal echocardiogram findings

, repaired structural congenital heart disease that now has otherwise normal echocardiogram findings

, history of a right ventricle to pulmonary artery conduit without stenosis or regurgitation of the pulmonary valve

, history of repaired atrioventricular canal defects without atrioventricular valve stenosis or regurgitation

, trace or mild regurgitation of any heart valve, bicuspid aortic valve without stenosis or regurgitation

, mild pericardial effusion, or patent foramen ovale with left to right shunting.

Examples of Clinically Significant Heart Disease include but are not limited to:

palliated heart disease that results in a single ventrical (e.g., history of Norwood, PDA stent, DKS, Glenn, or Fontan procedures), single ventricle physiology,

, any amount of depressed ventricular function

, patent foramen ovale with bidirectional or right to left shunting

, signs of pulmonary hypertension (e.g., RVSP or TR gradient > 25, ventricular septal flattening, or right ventricular hypertension)

, hypertrophic obstructive cardiomyopathy with or without obstructive findings

, any vascular stent with a stenosis gradient

, any type of mechanical circulatory support

, systemic or pulmonary venous stenosis gradients

, tetralogy of Fallot, double outlet right ventricle

, double inlet left ventricle

, ventricular septal defect

, atrial septal defect

, atrioventricular septal defect

, atrioventricular canal

, transposition of the great arteries

, hypoplastic left heart

, Ebstein’s anomaly

, pulmonary atresia

, William’s syndrome

, tricuspid atresia

, patent ductus arteriosus

, coarctation of the aorta

, anomalous pulmonary venous return (partial or total)

, truncus arteriosus, moderate to severe valvar stenosis

, moderate to severe valvar regurgitation or insufficiency

, or cardiac tumors.

If the patient has any Clinically Significant Heart Disease, then they are classified as having Clinically Significant Heart Disease even if the echocardiogram report contains other findings that are not clinically significant."""),},

{'role': 'user',

'content': ("Here are some example echocardiogram reports with clinician adjudication. "

+ **[INSERTED_TRAINING_ECHO_REPORT]**

+ ". Ignore the indication for the echocardiogram or any context irrelevant to findings of the exam. "

+ "If the patient had a heart transplant, ignore any other heart surgeries that would have occurred prior to the transplant including Norwood, Sano, Glenn, or Fontan procedures. "

+ "Do not use procedures that occurred prior to a heart transplant in consideration of whether a patient has Clinically Significant Heart Disease now. "

+ "Here is some chain of thought thinking process: "

+ **[INSERTED_CHAIN_OF_THOUGHTS]**

+ " Respond with a JSON in the format: "

+ '{ "Clinically Significant Heart Disease": "True"/"False" "bool // True or False. Determining whether the current echocardiogram report indicates a clinically significant diagnosis of any heart defect. "}')},

{'role': 'assistant',

'content': json.dumps({"Clinically Significant Heart Disease": **[INSERTED_ADJUDICATED_LABEL]**})}]

**Large Language Model (LLM) Testing Prompt**

[{'role': 'system',

'content': dedent(f"""System pretend you are a pediatric cardiovascular anesthesiologist.

You found the following content by searching through documentation. Use only this content to construct your response.

Think as a pediatric cardiovascular anesthesiologist and determine if the echocardiogram report content found is sufficient to classify if the patient has Clinically Significant Heart Disease,

classify if the following patient has Clinically Significant Heart Disease using the echocardiogram report

, and briefly explain the classification using medical terminology in no more than 50 words.

Ignore the indication for the echocardiograms, the quality of the exam, poor acoustic windows, technical limitations within the study, or any context irrelevant to findings of the exam.

If the patient had a heart transplant, ignore any other heart surgeries that would have occurred prior to the transplant including Norwood, Sano, Glenn, or Fontan procedures.

Do not use procedures that occurred prior to a heart transplant in consideration of whether a patient has Clinically Significant Heart Disease now.

Examples that would not qualify as Clinically Significant Heart Disease include but are not limited to:

a history of heart transplant with now otherwise normal echocardiogram findings

, repaired structural congenital heart disease that now has otherwise normal echocardiogram findings

, history of a right ventricle to pulmonary artery conduit without stenosis or regurgitation of the pulmonary valve

, history of repaired atrioventricular canal defects without atrioventricular valve stenosis or regurgitation

, trace or mild regurgitation of any heart valve, bicuspid aortic valve without stenosis or regurgitation

, mild pericardial effusion, or patent foramen ovale with left to right shunting.

Examples of Clinically Significant Heart Disease include but are not limited to:

palliated heart disease that results in a single ventrical (e.g., history of Norwood, PDA stent, DKS, Glenn, or Fontan procedures), single ventricle physiology,

, any amount of depressed ventricular function

, patent foramen ovale with bidirectional or right to left shunting

, signs of pulmonary hypertension (e.g., RVSP or TR gradient > 25, ventricular septal flattening, or right ventricular hypertension)

, hypertrophic obstructive cardiomyopathy with or without obstructive findings

, any vascular stent with a stenosis gradient

, any type of mechanical circulatory support

, systemic or pulmonary venous stenosis gradients

, tetralogy of Fallot, double outlet right ventricle

, double inlet left ventricle

, ventricular septal defect

, atrial septal defect

, atrioventricular septal defect

, atrioventricular canal

, transposition of the great arteries

, hypoplastic left heart

, Ebstein’s anomaly

, pulmonary atresia

, William’s syndrome

, tricuspid atresia

, patent ductus arteriosus

, coarctation of the aorta

, anomalous pulmonary venous return (partial or total)

, truncus arteriosus, moderate to severe valvar stenosis

, moderate to severe valvar regurgitation or insufficiency

, or cardiac tumors.

If the patient has any Clinically Significant Heart Disease, then they are classified as having Clinically Significant Heart Disease even if the echocardiogram report contains other findings that are not clinically significant."""),},

{'role': 'user',

'content': ("Here are some example echocardiogram reports with clinician adjudication. "

+ **[INSERTED_TRAINING_ECHO_REPORT]**

+ ". Ignore the indication for the echocardiogram or any context irrelevant to findings of the exam. "

+ "If the patient had a heart transplant, ignore any other heart surgeries that would have occurred prior to the transplant including Norwood, Sano, Glenn, or Fontan procedures. "

+ "Do not use procedures that occurred prior to a heart transplant in consideration of whether a patient has Clinically Significant Heart Disease now. "

+ "Here is some chain of thought thinking process: "

+ **[INSERTED_CHAIN_OF_THOUGHTS]**

+ " Respond with a JSON in the format: "

+ '{ "Clinically Significant Heart Disease": "True"/"False" "bool // True or False. Determining whether the current echocardiogram report indicates a clinically significant diagnosis of any heart defect. "}')}]
